# Trends in mortality and major adverse cardiovascular events following incident acute myocardial infarction

**DOI:** 10.1186/s12872-025-05481-2

**Published:** 2026-01-10

**Authors:** Steven Scholfield, Salwa S. Zghebi, Martin K. Rutter, Mamas A. Mamas, Evangelos Kontopantelis

**Affiliations:** 1 Division of Cardiovascular Sciences, School of Medical Sciences, Faculty of Biology, Medicine and Health, University of Manchester, Manchester, UK; 2 Division of Population Health, Health Services Research and Primary Care, School of Health Sciences, Faculty of Biology, Medicine and Health, University of Manchester, Manchester, UK; 3 Division of Diabetes, Endocrinology & Gastroenterology, School of Medical Sciences, Faculty of Biology, Medicine and Health, University of Manchester, Manchester, UK; 4 Keele Cardiovascular Research Group, Centre for Prognosis Research, Institute for Primary Care and Health Sciences, Keele University, Keele, UK; 5 Division of Informatics, Imaging and Data Sciences, School of Health Sciences, Faculty of Biology, Medicine and Health, University of Manchester, Manchester, UK

**Keywords:** Acute myocardial infarction, Mortality, Heart failure, Cerebrovascular accidents, Trends

## Abstract

**Background and aims:**

Assessment of mortality trends and real-world outcomes are important for monitoring acute myocardial infarction (AMI) care, although there is limited data beyond 1-year post incident AMI.

**Methods:**

We used Clinical Practice Research Datalink Aurum to identify patients ≥ 35 with incident AMI between 1 Jan 2006 to 31 Dec 2014. Data was also extracted from three other sources. Risk of all-cause and cardiovascular (CV)-related mortality, incident heart failure (HF), recurrent AMI, and cerebrovascular accidents (CVA) were calculated at 1- and 5-years using regression analysis—2006 was the comparator.

**Results:**

We identified 94,241 patients with AMI. The 1-year risk for multiple outcomes fell by 2014, including all-cause mortality (hazard ratio (HR): 0.82, 95% CI: 0.75-0.90), CV-related mortality (HR: 0.69, 95% CI: 0.60-0.78), and recurrent AMI (HR 0.72, 95% CI: 0.66-0.79). The 1-year risk for incident HF increased (HR: 1.18, 95% CI: 1.08-1.28) whilst CVA risk did not change (HR: 1.11, 95% CI: 0.98-1.26). At 5-years, the risk fell for all-cause mortality (HR: 0.82, 95% CI: 0.79-0.87), CV-related mortality (HR: 0.68, 95% CI: 0.62-0.74), and recurrent AMI (HR: 0.71, 95% CI: 0.65-0.75) by 2014. The 5-year risk for incident HF increased (HR: 1.15, 95% CI: 1.08-1.23), whilst CVA risk also increased significantly (HR: 1.16, 95% CI: 1.07-1.26) by 2014.

**Conclusions:**

Across 2006-2014, we observed a falling risk of all-cause mortality, CV-related mortality, and recurrent AMI at 1- and 5-years post incident AMI. Countervailing trends were seen for incident HF, where the risk for CVA also increased significantly by 5-years.

**Graphical Abstract:**

**Figure:**
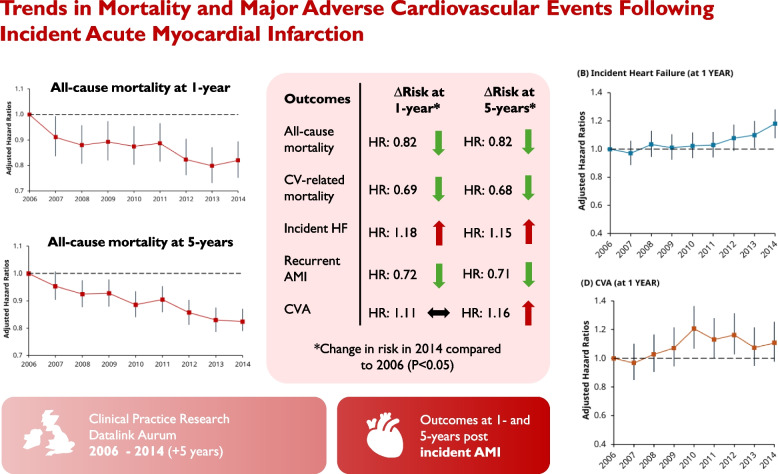

**Supplementary Information:**

The online version contains supplementary material available at 10.1186/s12872-025-05481-2.

## Introduction

Over the decades, landmark trials have shown effective strategies for the treatment of acute myocardial infarction (AMI) [1–3], leading to evolving practice guidelines around the world. However, in addition to assessing efficacy through randomised controlled trials, it is important to estimate treatment effect from real-world patient outcomes, including post AMI mortality and major adverse cardiovascular events (MACE).

Encouragingly, observational data has shown a decline in AMI mortality [4–11] and a variety of MACE subcomponents such as stroke [12–14] and recurrent AMI [10, 11]. However, not all MACE subcomponents have shown consistent trends in risk, including heart failure (HF) where the consensus is much more equivocal [5, 11, 15–18]. Moreover, much of the aforementioned data is limited to temporal trends at 1-year. This highlights a gap in the literature, as limited understanding beyond this interval prevents optimal benchmarking for services in acute and chronic disease management.

Therefore, to enhance understanding of short and longer-term patterns across this spectrum, providing longitudinal epidemiologic insights, we investigated the temporal 1- and 5-year trends of post-AMI mortality and MACE. This was performed in the UK between 2006 to 2014 using a nationwide real-life cohort.

## Methodology

### Settings and participants

All participants aged ≥ 35 years with incident AMI between 01/01/2006 to 31/12/2014 and no documented history of AMI before 2006, over the entire patient record, were included in the study.

### Data sources/measurement

We used the Clinical Practice Research Datalink (CPRD) Aurum, a comprehensive, longitudinal, and anonymised database capturing information from primary care centres throughout England in the United Kingdom (UK). As of 2018, this captured 7 million living patients (13% of the English population) across 738 practices (10% of English practices) [19]. We used CPRD Aurum to identify the relevant cohort with baseline characteristics including age, sex, body mass index (BMI), ethnicity, medications, smoking status, cardiometabolic disease, pulmonary disease, and renal disease that they had ever been diagnosed with before 2006. Notably, the database is also linked to other electronic healthcare records that allow for more comprehensive capture of patient data and outcomes. In this study, we used four other linked datasets as follows: the ‘Office for National Statistics’ was used to assess date of- and cause-specific death to differentiate between cardiovascular (CV) and non CV-related mortality; data from ‘Hospital Episode Statistics Admitted Patient Care’ (HES APC) was used to identify incident AMI and select MACE outcomes [20]; and data from the ‘Patient-Level 2011 Rural–Urban Classification at Lower Layer Super Output Area’ and the ‘Patient -Level Index of Multiple Deprivation’ (IMD) datasets were used to stratify participants by socioeconomic status [21, 22]. In England, IMD is the official measure for deprivation that is a composite score of 39 indicators across 7 domains on deprivation including income, health, and employment [22].

### Outcomes

The primary outcome assessed all-cause mortality at 1- and 5-years from the date of incident AMI (last follow up date hence being 31/12/2019, thereby avoiding the influence of the COVID pandemic). Secondary outcomes assessed CV-related mortality, incident heart HF, cerebrovascular accidents (CVA), and recurrent AMI as individual and composite outcomes (MACE) at 1- and 5-years. In supplementary analyses, the aforementioned were measured in hospital prior to discharge, and at 1- and up-to 5-years post discharge as a proxy for in-hospital and post-hospital care, respectively. For HES APC data, diagnoses/codes were defined as per the International Statistical Classification of Diseases and Related Health Problems 10th Revision (ICD-10, version 19) for each year [23] (Supplementary materials, Table S1), whilst those from primary care practices were defined using SNOMED read codes (Supplementary materials 2).

### Covariates

Clinically relevant covariates were assessed from baseline data. Statistical models were adjusted for age, sex, and a wider set of variables selected a priori. These included: BMI, coronary heart disease (CHD), ethnicity (White vs non-White), socioeconomic IMD, medication use (anticoagulants, antihypertensives, antiplatelets, and lipid lowering treatment), smoking status, diabetes, CVA, HF, hyperlipidaemia, chronic pulmonary disease, and chronic kidney disease (CKD).

### Statistical methods

Baseline characteristics were presented for all patients based on index year of AMI. Continuous data are presented as means ± standard deviation, and categorical variables are presented in frequency tables as percentages (or absolute counts if otherwise stated). Changes in trend for baseline characteristics across incident year of AMI were assessed using the Cochrane Armitage test for binary data, Jonckheere-Terpstra test for continuous variables, and linear-by-linear test for categorical variables with more than two categories.

Adjusted Cox regression models were used to determine the hazard of the primary and secondary outcomes at 1- and 5-years, stratified by the year of incident AMI as a categorical variable (2006 was the reference year), and this model was used due censored to its ability to handle data. Each Cox model was adjusted with covariates that were considered a priori and clinically relevant (described above). For these models, the proportional hazards assumption was checked using Schoenfeld residuals and log–log plots. Within each outcome-specific Cox model, patients were followed-up until the earliest date of the outcome of interest, censoring (leaving the general practice, last data collection date for the practice, study end date), or death (when appropriate) from their incident date. Baseline cases of HF were excluded from the Cox model for incident HF, and for the incident HF subcomponent of MACE outcomes.

Adjusted survival functions were obtained from the Cox model for the primary outcome and plotted across 1- and 5-years. Pre-defined subgroups and their influence on the risk for all-cause mortality were also highlighted, stratified by age, sex (male and female), socioeconomic status (by IMD quintiles), and ethnicity (White vs non-White) (Supplementary materials, Tables S2 and S3).

### Supplementary analyses

We performed multiple supplementary analyses. Competing risk models were performed for secondary variables using all-cause mortality as the competing risk. We also assessed if temporal trends in the primary and secondary outcomes were a function of changes across in-hospital or post-hospital care. To assess changes in-hospital, a proxy for in-hospital stay was derived from the time of the incident AMI to the closest date of discharge. Any event occurring within this time interval, including on the same day as discharge, was considered an in-hospital event. The odds of an event occurring in-hospital were then calculated across each incident year using an adjusted logistic regression model (2006 as the reference). To assess the hazards of post-hospital outcomes, landmark sensitivity analyses were performed using an adjusted Cox model at 1- and up to 5-years from the date of discharge (landmark date) in patients who were event-free (at discharge) across each year (2006 as the reference). As our total follow time was limited to 5-years from the incident date of AMI, all patients who had an inpatient stay will have had a follow time from discharge of 5-years minus this stay. Hence, follow up could only be considered up to 5-years, where the length of the inpatient stay was further adjusted for as a covariate to account for this. Further, to determine the relative contributions of changes across in-hospital and post-discharge mortality rates on total mortality, we performed a counterfactual decomposition analysis (conceptually akin to a Blinder–Oaxaca decomposition [24]). This technique divided the total change in mortality into segments (inpatient- and post-discharge mortality), and modelled changes in mortality under counterfactual conditions for each segment. This method is further detailed in our Supplementary materials. For all analyses, risks and odds are presented as hazard ratios (HRs) and odds ratios (ORs) with 95% confidence intervals (CI). Statistical significance was considered at *P* < 0.05. Stata v17. (StataCorp, Texas, USA) was used for statistical analysis.

### Missing data

Missing data for baseline characteristics were handled through multiple imputation, with an imputation model that included all covariates of interest and closely matched the analytical model. Imputed data was not used when constructing adjusted Cox and logistic regression models, as to avoid imputation for an outcome of interest (CV-related death, etc.) when such data was missing.

## Results

### Patient characteristics

We identified 94,241 patients aged 35 years or older who were eligible for inclusion between 01/01/2006 to 31/12/14. Table 1 shows that some clinical characteristics of patients with incident AMI were different across the years. Over time, patients were less likely to be White or female. The prevalence of cardiac disease and other conditions did not change uniformly with some diagnoses becoming more prevalent (i.e., CKD) and some becoming less prevalent over time (i.e., CHD). The baseline prescriptions of cardiac-specific medications (prior to the index AMI), including anticoagulants, antihypertensives, antiplatelets, and lipid-lowering therapy, all increased with time (Table 1).

**Table 1:** Baseline characteristics of patients with incident acute myocardial infarction

**Characteristic**	2006	2007	2008	2009	2010	2011	2012	2013	2014	*P*-Value (Trend)
No. of Patients	10,153	10,042	10,387	10,278	10,475	10,805	10,985	10,641	10,475	
Age – years (**± **SD**)**	69 ± 13	69 ± 14	70 ± 14	69 ± 14	70 ± 14	70 ± 14	70 ± 14	70 ± 14	69 ± 14	0.812
Sex (Male), %	62	62	62	63	62	63	63	63	64	0.003
Body Mass Index (**± **SD)	27 ± 5.3	27 ± 5.4	27 ± 5.4	28 ± 5.4	28 ± 5.5	28 ± 5.5	28 ± 5.5	28 ± 5.6	28 ± 5.6	< 0.001
Ethnicity, %										< 0.001
White	94	94	93	93	93	93	93	92	92	
Non-White	5.6	6.4	6.7	7.2	7.2	7.0	7.2	8.3	8.3	
IMD, %										0.116
Q1 (Least deprived)	20	20	20	20	21	20	20	21	20	
Q2	20	20	21	20	20	21	21	21	21	
Q3	20	20	20	21	20	21	20	20	20	
Q4	20	20	20	19	19	19	20	20	20	
Q5 (Most deprived)	20	20	20	20	20	19	19	19	20	
Medication Use, %										
Anticoagulants	5.3	5.9	6.2	6.2	6.9	7.4	7.8	7.9	7.5	< 0.001
Antihypertensives	69	69	69	68	70	70	70	70	70	< 0.001
Antiplatelets	44	45	47	47	47	47	47	46	45	0.036
Lipid-lowering	39	42	45	47	50	50	52	53	53	< 0.001
Alcohol status, %										0.106
Drinks alcohol	82	82	82	82	82	83	83	82	83	
Non/former drinker	18	18	18	18	18	17	17	18	17	
Smoking, %										< 0.001
Current Smoker	31	31	32	33	33	32	33	33	33	
Ex-Smoker	49	49	49	49	49	50	49	49	49	
Never Smoked	20	20	19	18	18	18	19	18	18	
Cardiac Disease, %										
Coronary heart dis	30	29	28	27	27	27	26	25	25	< 0.001
Hyperlipidaemia	16	17	18	18	20	20	21	22	22	< 0.001
Heart failure	7.0	6.6	6.3	6.5	6.0	5.8	6.1	5.7	5.7	< 0.001
Hypertension	39	40	40	41	42	42	42	43	42	< 0.001
Other Conditions, %										
Chronic kidney dis	8.6	17	19	20	21	21	21	21	20	< 0.001
Chronic resp. dis	12	12	13	13	13	14	14	14	13	< 0.001
Diabetes	17	17	17	18	19	19	19	21	21	< 0.001
CVA/TIA	9.7	9.8	9.9	10	11	11	10	10	9.9	0.064

Baseline characteristics of patients with incident AMI by year. No. of patients is presented as absolute totals by year. *P*-values are represented to 3 decimal places. All other results are expressed to 2 significant figures except for ‘No. of Patients’ that is presented as absolute counts

*CHD* coronary heart disease, *CKD* chronic kidney disease, *CV* cardiovascular, *CVA* cerebrovascular accident, *IMD* index of multiple deprivation, *PVD* peripheral vascular disease, *SD* standard deviation, *TIA* transient ischaemic attack, *Q* quintile

### All-cause mortality

In total there were 9,432 patients who died from any cause at 1-year post incident AMI and 24,410 patients who died at 5-years. The respective annual distributions are shown in Table 2. Comparing the start and the end of the observation period (2006 to 2014), the proportion of patients who died from any cause decreased at both 1- and 5-years. In each instance, the absolute reduction from 2006 to 2014 was approximately 1% (11 to 10% at 1-year, and 30 to 29% at 5-years).

**Table 2:** Proportions of patients with the primary and secondary outcomes by incident year

**Outcomes**	2006	2007	2008	2009	2010	2011	2012	2013	2014	*P*-Value (Trend)
All-Mortality										
1-Year	11	11	11	11	11	11	10	10	10	0.006
5-Years	30	30	31	31	30	31	30	29	29	0.003
CV-Mortality										
1-Year	5.3	4.7	4.8	4.8	4.3	4.0	3.9	3.7	4.0	< 0.001
5-Years	11	11	11	10	9.8	9.0	8.7	8.3	8.5	< 0.001
Heart Failure										
1-Year	16	16	17	18	18	19	19	21	21	< 0.001
5-Years	22	22	24	24	26	26	26	28	28	< 0.001
Recurrent AMI										
1-Year	11	12	12	11	9.5	8.1	9.0	8.4	8.6	< 0.001
5-Years	15	15	15	14	12	11	11	11	11	< 0.001
CVA										
1-Year	6.6	6.6	7.0	7.6	8.7	8.1	8.3	8.1	8.0	< 0.001
5-Years	12	13	13	14	15	15	15	15	15	< 0.001
MACE										
1-Year	34	34	35	35	35	34	35	35	36	0.037
5-Years	46	46	47	47	47	46	46	47	47	0.068

Descriptive statistics in the left-hand columns (from 2006 to 2014) showing the absolute percentage of persons who had experienced the relevant primary or secondary outcome at 1- and 5-years based on year of incident AMI. MACE was a composite of CV-related mortality, incident HF, CVA, and recurrent AMI. The right-hand column shows the P-Value for trend using Cochrane Armitage tests

*AMI* acute myocardial infarction, *CI* confidence interval, *CV* cardiovascular, *CVA* cerebrovascular accident, *HR* hazard ratio, *MACE* major adverse cardiovascular event. Results are presented to two significant figures

^*^Significance value of *P* < 0.05. Outcome specific *P*-values can be seen in Supplementary Materials

**Fig. 1:**
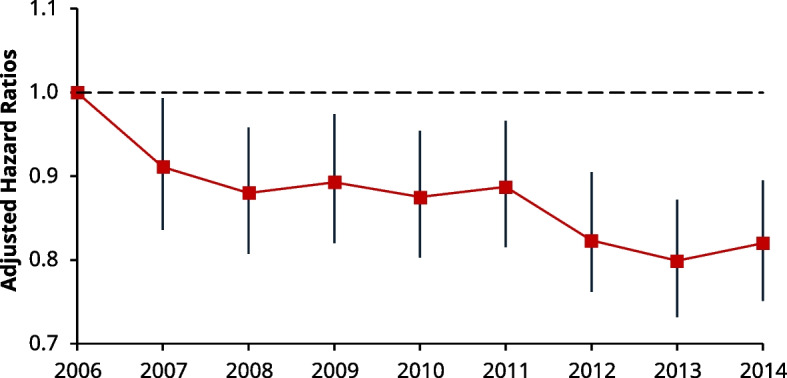
Line chart of the adjusted hazard ratios (HRs) for 1-year all-cause mortality by incident year using Cox regression. Each HR is compared to the reference (2006) with I bars representing 95% confidence intervals (CI) shown above and below. By ascending year, the respective HRs are: 2007 (HR: 0.91, 95% CI: 0.84–0.99), 2008 (HR: 0.88, 95% CI: 0.81–0.96), 2009 (HR: 0.89, 95% CI: 0.82–0.97), 2010 (HR: 0.88, 95% CI: 0.80–0.95), 2011 (HR: 0.89, 95% CI: 0.82–0.97), 2012 (HR: 0.82, 95% CI: 0.76–0.91), 2013 (HR: 0.80, 95% CI: 0.73–0.87), and 2014 (HR: 0.82, 95% CI: 0.75–0.90). ^*^Significance value of *P* < 0.05

The adjusted Cox model for 1-year all-cause mortality showed the risk of death changed at each year of incident AMI, decreasing over time (adjusted HRs compared with the reference year: 2007 [HR 0.91, 95% CI: 0.84–0.99] and 2014 [HR: 0.82, 95% CI: 0.75–0.90]) (Fig. 1).

**Fig. 2:**
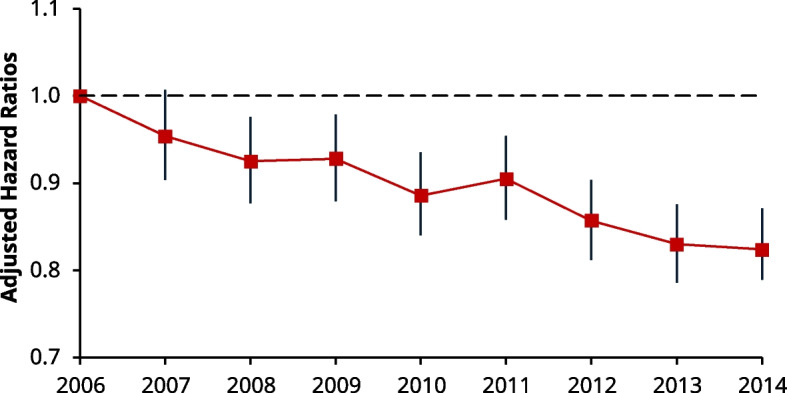
Line chart of the adjusted hazard ratios (HRs) for 5-year all-cause mortality by incident year using Cox regression. Each HR is compared to the reference (2006) with I bars representing 95% confidence intervals (CI) shown above and below. By ascending year, the respective HRs are: 2007 (HR: 0.95, 95% CI: 0.90–1.01), 2008 (HR 0.93, 95% CI: 0.88–0.98), 2009 (HR: 0.93, 95% CI: 0.88–0.98), 2010 (HR: 0.89, 95% CI: 0.84–0.94), 2011 (HR: 0.91, 95% CI: 0.86–0.95), 2012 (HR 0.86, 95% CI: 0.81–0.90), 2013 (0.83, 95% CI: 0.79–0.88), and 2014 (HR: 0.82, 95% CI: 0.78–0.87)

Similar to 1-year mortality, the same trend was seen in the adjusted Cox model for all-cause mortality at 5-years. Although the risk of death was not statistically different in 2007 compared to 2006 (HR 0.95, 95% CI: 0.90 to 1.01), the HRs gradually fell thereafter to a nadir in 2014 (HR 0.82, 95% CI: 0.79 to 0.87) (Fig. 2).

**Fig. 3:**
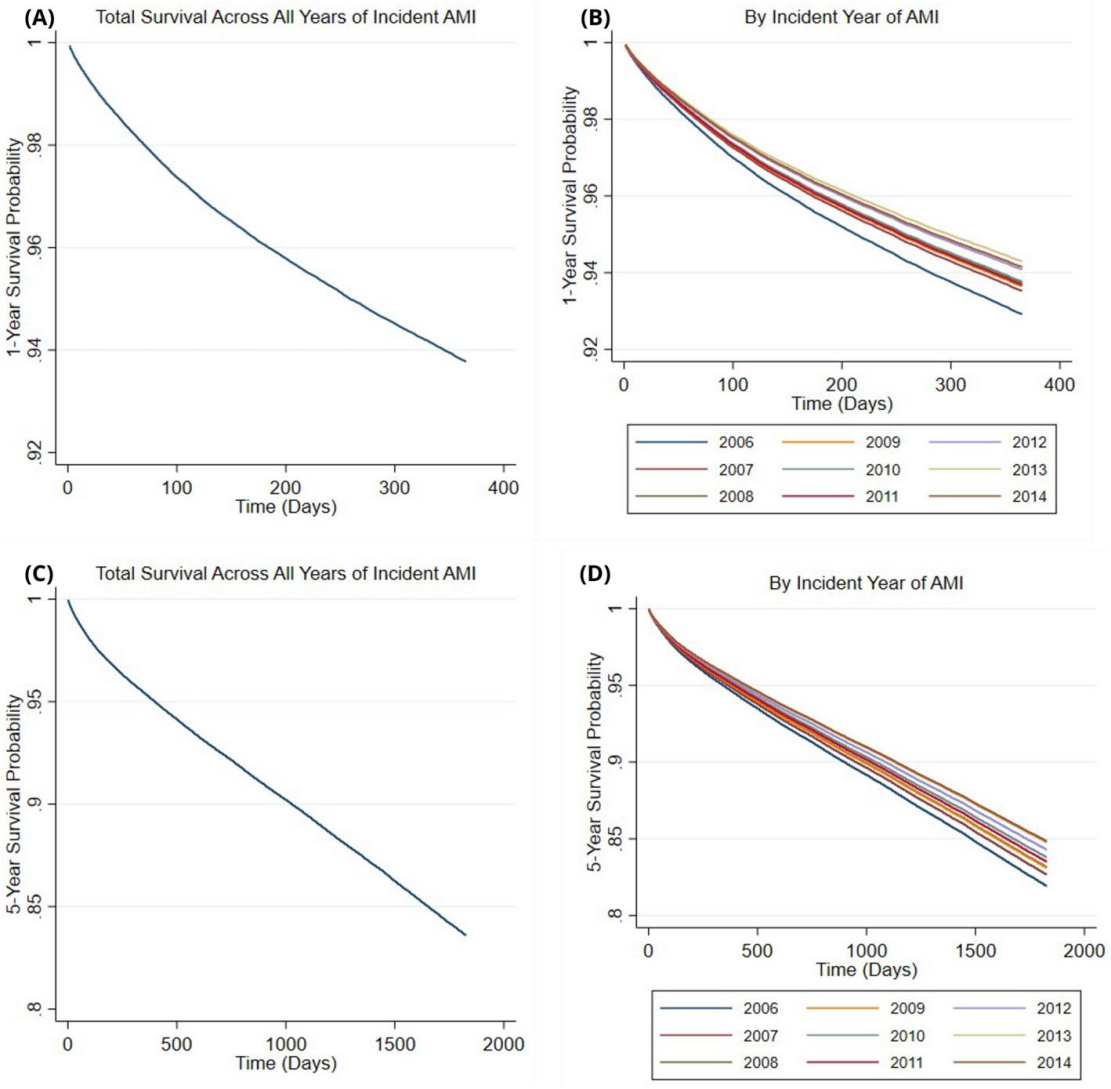
Cox proportional hazards regression models of the survival probabilities at 1- and 5-years post incident acute myocardial infarction (AMI). **A** (top left): The 1-year survival probability of all persons who had an incident AMI during the study period, regardless of the year of incident AMI. **B** (top right): The 1-year survival probability of all persons who had an incident AMI during the study period, stratified by the year of incident AMI. **C** (bottom left): The 5-year survival probability of all persons who had an incident AMI during the study period, regardless of the year of incident AMI. **D** (bottom right): The 5-year survival probability of all persons who had an incident AMI during the study period, stratified by the year of incident AMI. For each 1-year survival graph (**A** and **B**), the total follow-up time terminates at 365 days (1-year). For each 5-year survival each graph (**C** and **D**), the total follow-up time terminates at 1825 days (5-years). The incident year of AMI was inputted as a categorical variable within the Cox regression model to generate the above survival curves

In Fig. 3, the mean survival probabilities, show a gradual decrease with time across 1- and 5-years (Fig. 3A and C). The slope of each mean survival curve is most pronounced in the immediacy post AMI, suggesting the probability of death is highest at this point. Subsequently, the mean 5-year survival curve (Fig. 3C) begins to decrease linearly at 1-year, suggesting the probability of death stabilises at this point. The survivor functions for 1- and 5-years by incident year of AMI are shown in Fig. 3B and D, respectively. The probability of death at any time post AMI is highest in 2006 and lowest at later years (2012 to 2014) across the entirety of the 5-year follow up.

### Cardiovascular mortality

**Fig. 4:**
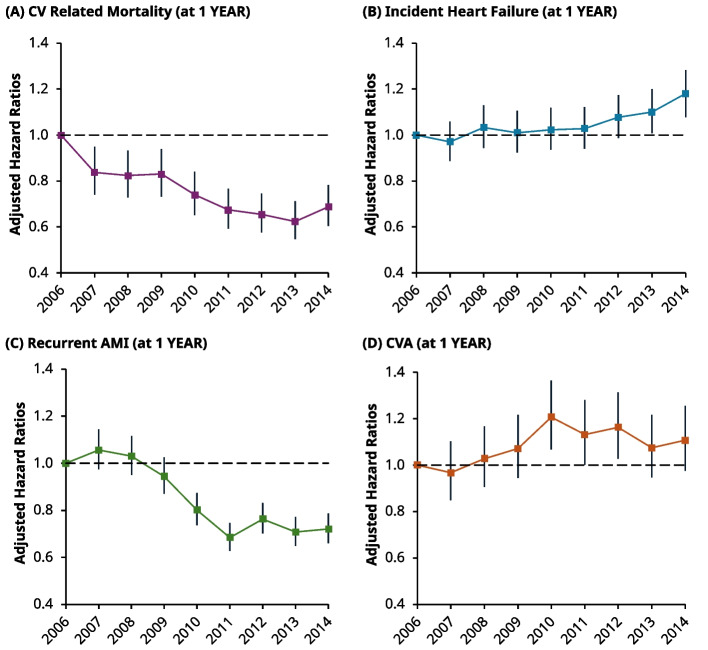
Line charts of the adjusted hazard ratios (HRs) for individual secondary outcomes at 1-year post incident year excluding MACE events, using Cox regression. Each HR is compared against 2006 as the reference in all graphs, with corresponding 95% CIs. *Top Left *(**A**): Risk of CV-related mortality, 2007: (HR 0.84, 95% CI 0.74–0.95), 2014: (HR: 0.69, 95% CI: 0.60–0.78). *Top Right *(**B**): Risk related to incident heaty failure post event: 2007 (HR: 0.97, 95% CI: 0.89–1.06) and raised risk in 2014: (HR: 1.18, 95% CI: 1.08–1.28). *Bottom Left *(**C**): Risk of recurrent AMI event post incident event. In 2007, the risk gradually decreased with time from approximately 2009, before reaching a nadir in 2011–2007 (HR: 1.06, 95% CI: 0.98–1.14) and 2014 (HR 0.72, 95% CI: 0.66–0.79) *Bottom Right *(**D**): Risk of a CVA (i.e., stroke, TIA) event. 2007 (HR: 0.97, 95% CI: 0.85–1.102) and 2014 (HR: 1.11, 95% CI: 0.98–1.26)

**Fig. 5:**
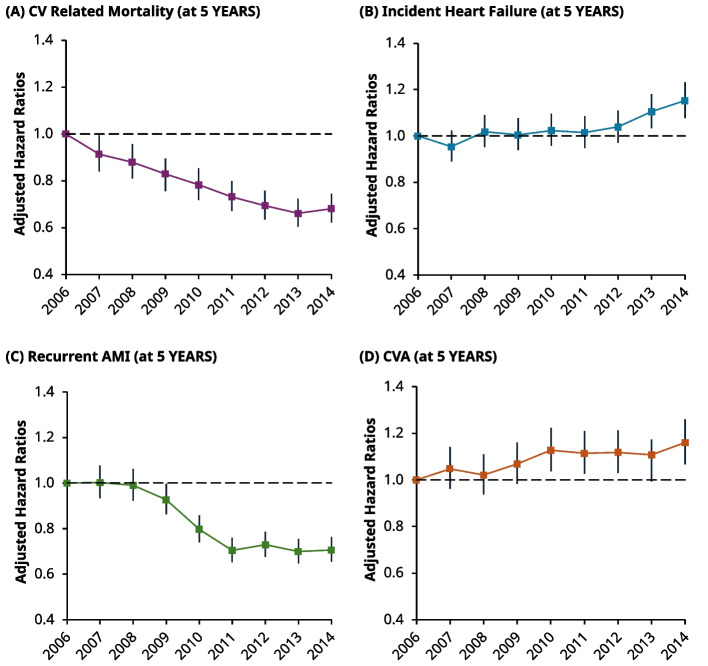
Line chart of the adjusted hazard ratios (HRs) for individual secondary outcomes at 5-years post incident year, excluding MACE events, with corresponding 95% CI’s using Cox regression. *Top Left *(**A**): Risk of CV-related mortality, 2007 (HR: 0.91, 95% CI: 0.84–0.99) and 2014 (HR: 0.68, 95% CI: 0.62–0.74). *Top Right *(**B**): Risk of Incident HF, 2007 (HR: 0.95, 95% CI: 0.89–1.02) and 2014 (HR: 1.15, 95% CI: 1.08–1.23). *Bottom Left *(**C**)*:* Risk of Recurrent AMI, 2007 (HR: 1.00, 95% CI: 0.93–1.08) and 2014 (HR: 0.71, 95% CI: 0.65–0.75). *Bottom Right *(**D**): Risk of CVA, 2007 (HR: 1.05, 95% CI: 0.96–1.14) and 2014 (HR: 1.16, 95% CI: 1.07–1.26)

Together, a total of 4,118 and 9,058 patients had died from a CV-related event at 1- and 5-years, respectively. Compared with all-cause mortality, similar trends were seen in the proportion of people experiencing CV-related death. Namely, the proportion of CV-related deaths at 1 and 5-years fell by 1.3% (5.3% to 4%) and 2.5% (11% to 8.5%) from 2006 to 2014, respectively (Table 2). These trends are corroborated by the fall in risk from adjusted 1-year Cox models; compared to 2006, the risk of CV-related death fell by 16% (HR: 0.84, 95% CI: 0.74–0.95) and 31% (HR: 0.69, 95% CI: 0.60–0.78) in 2007 and 2014, respectively (Fig. 4, top left). Moreover, the 5-year risk of CV-related death fell monotonically with each incident year, reaching a nadir in 2013 (HR: 0.66, 95% CI: 0.61–0.72) (Fig. 5, top left).

### Incident heart failure

At 1-year, there were 20,864 patients who were coded as having developed incident HF. However, after excluding patients in this group who had a concurrent baseline diagnosis of HF (3,688 patients), this left a total of 17,176 patients who developed incident HF at 1-year. The risk of developing HF remained similar up to 2011 (compared to 2006). However, from 2012, the HRs gradually increased before becoming statistically significant in 2013 (HR: 1.10, 95% CI: 1.01–1.20) and reaching their peak in 2014 (HR: 1.18, 95% CI 1.08–1.28) (Fig. 4, top right). At 5-years, there were 28,20 patients who were coded as having developed incident HF. However, after excluding patients in this group who had a concurrent baseline diagnosis of HF (4,501 patients), this left 23,703 patients who developed incident HF at 5-years.The HRs at 5-years followed a related pattern to 1-year, with the risk similar up to 2012, before significantly increasing at 2013 (HR: 1.11, 95% CI: 1.03–1.18) and peaking in 2014 (HR: 1.15, 95% CI: 1.08–1.23) (Fig. 5, top right).

### Recurrent AMI

At 1-year, a total of 9,432 patients experienced recurrent AMI, although the proportion of events was higher in 2006 (11%) compared to 2014 (8.6%) (Table 2). The same trend was seen at 5-years post incident AMI (15% in 2006 compared to 11% in 2014). Adjusted Cox models showed that the risk at 1-year started to decline from 2009 sharply to a nadir in 2011 (HR: 0.68, 95% CI: 0.63–0.75), before this risk appeared to plateau up-to-and-including 2014 (HR: 0.72, 95% CI: 0.66–0.79) (Fig. 4, bottom left). The risk at 5-years also fell sharply after 2009, before stabilising at 2011 (HR: 0.70, 95% CI: 0.65–0.76) (Fig. 5, bottom left).

### Cerebrovascular accidents

In total there were 7,241 patients who had experienced a CVA event at 1-year post incident AMI, whilst a total of 13,321 patients had experienced an event at 5-years. The proportion of events, from 2006 to 2014, increased from 6.6% to 8.0% at 1-year and 12% to 15% at 5-years, respectively (Table 2). The risk at 1-year was statistically higher in 2010 to 2012 (HR: 1.21, 95% CI: 1.07–1.37) (Fig. 4, bottom right), but not across other years. At 5-years, the risk for CVA generally increased over time, becoming statistically higher by 2014 (HR 1.16, 95% CI: 1.07–1.26) (Fig. 5, bottom right).

### Major adverse cardiovascular events

**Fig. 6:**
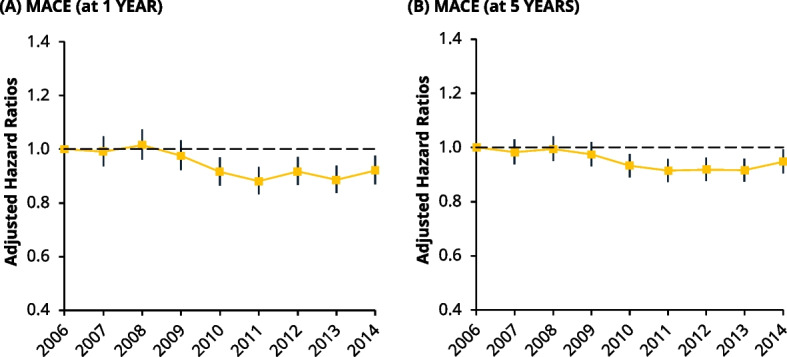
Line chart of the adjusted hazard ratios (HRs) for MACE outcomes at 1- and 5-years post incident year using Cox regression. *Left *(**A**): Risk of MACE at 1-year, 2007 (HR: 0.99, 95% CI: 0.94–1.05) and 2014 (HR: 0.92, 95% CI: 0.87–0.98). *Right *(**B**): Risk of MACE at 5-years, 2007 (HR: 0.98, 95% CI 0.94–1.03) and 2014 (HR: 0.95, 95% CI: 0.90–0.99)

The MACE event rate was calculated from a composite of CV-related death, incident HF, recurrent AMI, and CVA. At 1-year and 5-years, there were a total of 32,717 and 43,903 events, respectively. Proportionally, from 2006 to 2014, MACE events increased by 2% (34% to 36%) at 1-year and 1% (46% to 47%) at 5-years, respectively (Table 2). Adjusted Cox models showed that the risk of MACE at 1-year started to decrease linearly after 2009, before plateauing to 2014 (HR: 0.91, 95% CI: 0.86–0.97) (Fig. 6, left). At 5-years, a similar patten was seen, where the risk of a fell after 2009, before stabilising through to 2014 (HR: 0.94, 95% CI: 0.89–0.98 (Fig. 6, right).

### Supplementary analysis (Competing Risk Models)

Competing risk models were performed using a Fine and Gray risk regression model [25] to determine the sub-distribution hazard ratio (SHR) of secondary outcomes when all-cause mortality acted as a competing risk. Across 1- and 5-year outcomes, similar trends were observed in both the competing risk and HRs across Cox models for each secondary outcome (i.e., falling SHR and HR for CV-related death, respectively). Full outputs and trends are show in Supplementary Materials (Table S24 to S31).

### Supplementary analysis (Mortality and Non-Fatal Outcomes After Discharge)

To further examine the relationship between mortality and non-fatal CV outcomes with incident year of AMI, supplementary analyses were performed to assess if the temporal trends in the aforementioned results were due to disparate improvements in post-hospital or in-hospital outcomes. Adjusted Cox models were used to perform a landmark sensitivity analysis of outcomes with 1- and up to 5-years follow up from the date of discharge rather than the incident AMI. At each follow up time, the proportions and risk of all-cause mortality, CV-related mortality, and recurrent AMI decreased (*P* < 0.05 for comparison of 2014 with 2006) (Table 3). Countervailing trends were seen in incident HF and CVA, where the proportion of persons having an event, and the risk of each event at 1- and up to 5-years had increased (*P* < 0.05 for comparison of 2014 with 2006) (Table 3). Contrasted to how the risk of MACE events fell over time in the primary Cox model at 1- and 5-years (Fig. 6), the risk of MACE events at 1- and 5-years did not significantly change with each year of AMI in the discharged group (*P* > 0.05) (Table 3). For each year across all the models described above, their HRs (compared to 2006) can be seen in the supplementary materials (Tables S4 to S15).

**Table 3:** Primary and secondary outcomes in patients discharged alive from hospital

**Outcomes**	Hazard ratio 2007 (vs. 2006)	Hazard ratio 2014 (vs. 2006)
All-Cause Mortality		
1-Year	0.91 (0.83–0.99)	0.83 (0.75–0.90)
Up to 5-Years	0.95 (0.90–1.00)	0.82 (0.78–0.87)
CV-Related Mortality		
1-Year	0.81 (0.71–0.93)	0.69 (0.60–0.79)
Up to 5-Years	0.91 (0.83–0.99)	0.68 (0.62–0.75)
Incident HF		
1-Year	0.94 (0.85–1.03)	1.19 (1.09–1.31)
Up to 5-Years	0.93 (0.87–1.00)	1.16 (1.08–1.24)
Recurrent AMI		
1-Year	0.99 (0.86–1.13)	0.80 (0.70–0.93)
Up to 5-Years	0.92 (0.83–1.02)	0.74 (0.67–0.83)
CVA		
1-Year	0.94 (0.82–1.07)	1.11 (0.98–1.26)
Up to 5-Years	1.03 (0.95–1.12)	1.14 (1.05–1.24)
MACE		
1-Year	0.93 (0.86–1.00)	1.04 (0.97–1.12)
Up to 5-Years	0.95 (0.90–1.00)	1.02 (0.97–1.08)

Adjusted hazard ratios for the risk of primary and secondary outcomes at 1- and 5-years in people discharged alive from hospital in 2007 and 2014, compared to 2006. The middle column compares risk of each outcome in the most immediate year (2007) after the reference year (2006) of incident AMI, whilst the right-hand column compares each risk at the end of the study period for incident AMI (2014) on the reference year. For 5-year outcomes, follow up time up to 5-years was used from the date of discharge, as the maximum follow up time available was 5-years from the date of the incident event and not discharge. Cox models were also adjusted for in hospital stay in adjusted Cox models up to 5-years

*AMI* acute myocardial infarction, *CI* confidence interval, *CV* cardiovascular, *CVA* cerebrovascular accident, *HR* hazard ratio, *MACE* major adverse cardiovascular event (composite of CV-related mortality, incident HF, recurrent AMI and CVA). Statistical significance at P < 0.05

### Supplementary analysis (Odds of In-Hospital Events Over Time)

Alongside the risk of events occurring post-discharge, a further supplementary analysis was performed to assess how the odds of the primary or secondary outcomes changed over time whilst patients were still in hospital, as a proxy of inpatient care. Adjusted logistic regression models showed that across each outcome (primary and secondary), the odds of any event decreased over time and were significantly lower among patients who had their incident AMI in 2014 compared to 2006 (*P* < 0.05) (Table 4). For the interval years between, the odds ratios (ORs) for all outcomes decreased with each advancing year (Supplementary materials, Table S16 to S21).

**Table 4:** Odds of in-hospital outcomes prior to discharge over time

**Outcomes**	Odds ratio 2007 (vs. 2006)	Odds ratio 2014 (vs. 2006)
All-Cause Mortality	0.83 (0.71–0.97)	0.36 (0.30–0.44)
CV-Related Mortality	0.86 (0.67–1.10)	0.40 (0.29–0.54)
Incident HF	0.95 (0.88–1.04)	0.70 (0.64–0.76)
Recurrent AMI	0.96 (0.87–1.05)	0.45 (0.41–0.51)
CVA	0.94 (0.84–1.04)	0.56 (0.50–0.63)
MACE	0.96 (0.90–1.02)	0.57 (0.54–0.61)

Adjusted odds ratios (ORs) for the odds of the primary and secondary outcomes occurring whilst still in hospital, prior to discharge, based on the incident years for AMI of 2007 and 2014, compared to 2006. The ORs represent the odds of an event occurring based on how the proportion of in-hospital events for each outcome changed as a proportion of the whole population at risk for each incident year

*AMI* acute myocardial infarction, *CI* confidence interval, *CV* cardiovascular, *CVA* cerebrovascular accident, *HR* hazard ratio, *MACE* major adverse cardiovascular event (composite of CV-related mortality, incident HF, recurrent AMI and CVA). Statistical significance at *P* < 0.05

### Supplementary analysis (Counterfactual Decomposition Analysis of Mortality)

A further supplementary analysis was performed to determine the relative contributions of changes in in-hospital and post-discharge mortality on total mortality by 2014. This demonstrated that, compared to 2006, the absolute change in 1-year all-cause mortality was −6.0%, and 72% of this change was attributable to changes across in-hospital mortality, whilst 28% were due to changes in post-discharge mortality (Supplementary materials, Table 22 to 23).

## Discussion

This observational study found 94,241 independent events of incident AMI in the UK across 2006 to 2014. The risk for 1- and 5-year mortality (all-cause and CV-related), recurrent AMI, and composite MACE decreased over time from 2006 to 2014, whilst a countervailing trend was seen for rates of HF and stroke.

### All-cause and cardiovascular-related mortality

We found that the risk for all-cause and CV-related mortality at 1-year decreased over time from 2006 to 2014. Due to the observational study design, we are unable to causally explain our results. However, our results provide longitudinal epidemiologic insights that are consistent with mortality trends across post AMI-studies [4–11], where there were prevalent changes in revascularisation strategies, pharmacotherapy, and diagnostic strategies across the UK.

At the turn of the millennium, primary percutaneous coronary intervention (PCI) demonstrated superior CVD endpoints (including death, non-fatal AMI, and stroke) compared to thrombolysis up to 2-h post symptom onset [1–3]. This paradigm prompted a UK-government funded feasibility study in 2004 to evaluate the effectiveness of transitioning to PCI as the primary treatment for AMI [26], and subsequent outcomes drove a significant surge in the UK’s use of PCI. Specifically, national PCI use across eligible patients increased from 46% in 2008 to 95% by 2012 [26, 27]. As our cohort was likely captured in this national expansion, such changes across PCI use likely influenced the survival trends we saw.

Over our study period, the use of dual antiplatelet therapy (DAPT) changed across the UK, and this may also explain the lower risk of death we observed. Landmark trials such as TRITON-TIMI 38 and PLATO demonstrated that prasugrel and ticagrelor were superior to clopidogrel in reducing adverse cardiac events post AMI [28, 29]. In each instance, this was part of a DAPT strategy with aspirin. Consequently, in the early 2010’s, international guidelines began to advocate for a transition away from using clopidogrel in DAPT [30, 31]. This likely influenced the shift toward using ticagrelor that occurred in the UK during the same time, particularly for patients undergoing PCI [32].

Moreover, survival may have also been influenced by secular trends in the routine use of high-sensitivity troponin as a diagnostic tool for AMI [33], introduced in Europe in 2010 [34] and increasingly used within the UK thereafter [35]; this likely led to earlier detection and management of more subtle infarcts in the in-hospital setting, contributing to a greater survival outcome for patients in the latter half of our study.

Of note, the relative risk for 5-year all-cause and CV-related mortality decreased during the study period. Interrogation of our survival curves suggests that the greatest change between incident years on mortality occurred in the immediacy after AMI, with the probability stabilising at 1-year thereafter. As such, this suggests that acute phase care (rather than longer term care) likely had a greater impact on the trends in mortality we observed, despite proxy improvements across in-hospital and post-discharge care (due to falling odds and risk of in-hospital and post-discharge mortality at 1-year, respectively). Decomposition analysis of the relative contributions further supported this notion, as 72% of the fall in the all-cause mortality rate (at 1-year in 2014 compared to 2006) was due to improvements across in-hospital mortality and hence acute patient care.

Contextually, changes in acute care would be explained by the aforementioned changes in PCI use; follow up data from the landmark PRAGUE-2 trial showed that any mortality benefit seen at 5-years with PCI was due to a reduction in mortality within the first 30 days post-intervention [36]. Similarly, any benefit from DAPT therapy for all-cause mortality appears to attenuate after 12-months, owing to an increased risk of non-CV mortality (i.e., major bleeding) that offsets any reduction in CV-related mortality [37]. Indeed, survival also appears to improve after 12-months between incident years, perhaps through greater use of secondary prevention therapies (i.e., ACE inhibitors) or due to the downstream effects of more effective acute treatment. However, as the greatest benefit in survival appears largely attributable to better short-term outcomes, this highlights the importance of high-quality early intervention and benchmarks the long-term value of optimising early intervention strategies, both at the clinical and policy level.

### Recurrent acute myocardial infarction

The relative risk for recurrent AMI at 1- and 5-years significantly decreased during the study period. Mechanistically, the reasons for this are likely related to the processes that also reduced mortality post AMI. There was also an increase in the use of drug eluting stents in the UK during the study period, and these were associated with a decreased risk of stent restenosis and thrombosis compared to bare metal stents [38]. If our later-year patient cohorts were subject to changes in stent design, this may further explain the decreased risk we observed.

### Heart failure

Our findings suggest that the temporal risk of incident HF increased at 1- and 5-years post AMI. In contrast, many studies have seen a reduction in risk, incidence, or prevalence over time. National analysis of first-time AMI hospitalisation in Denmark between 1997 to 2010 reported a lower risk for incident HF at 90-days, with a HR of 0.82 (*P* < 0.001) at 2010 compared to 1997 [15]. Similarly, another Danish study using national hospitalisation data from 2000 to 2017 showed the risk of first-time HF admission (post discharge from incident AMI) fell at 1-year by the study end (HR: 0.82, *P* < 0.001) [5]. Related observations are also reported in New Zealand and Sweden. Using trend analysis between 2006 to 2016, Wang et al. reported a year-on-year OR of 0.982 (*P* < 0.001) for HF hospitalisation post incident AMI [11], whilst a national Swedish register-based analysis of first-time AMI hospitalisation (between 1993 to 2004) reported a 4% (*P* < 0.001) year-on-year risk reduction for developing incident HF at 3-years [17]. Indeed, these observations are not concordant with our findings, but this may be related to the aforementioned solely measuring HF hospitalisation that would have not captured HF diagnoses that were also occurring in the community.

Our use of a linked CPRD dataset would have captured any increase in community-based HF diagnoses; by 2010 in the UK, early referral for suspected HF post-AMI became standardised to specialist community services, and any increase in diagnoses thereafter would not have been included in hospitalisation data [39]. This concept is supported by our observation of rising risk for incident HF, despite the falling in-hospital odds each year, suggesting that the former was influenced by changes in the post-discharge setting. Indeed, our results may also be due to competing risks and survivorship bias, a notion supported by data from the UK’s Myocardial Ischemia National Audit Project (MINAP); amongst all 1,046,480 patients admitted with AMI between 2006 to 2019, the 1-year incidence of HF hospitalisation generally increased with each year, despite a fall in mortality [16]. Importantly, MINAP *only* captured re-hospitalisation data and hence likely underestimated the true spectrum of disease across UK; our estimates are likely more reflective of current landscape. Nonetheless, regardless of the definitive cause, our results highlight that the burden of post-AMI HF was an important historical consideration during our study period.

### Cerebrovascular accidents

We observed a significant increase in the risk for CVA post-AMI at 5-years, with a non-significant trend for a higher 1-year risk also seen over time. These observations appear to contradict the steady decline in stroke (both ischemic and haemorrhagic) observed in the broader UK population [40]. The disparity could be partially due to improved survival, resulting in a larger at-risk population for subsequent complications associated with CVA, including atrial fibrillation.

Another contributing factor may be explicated through our definition of CVA. As well as haemorrhagic and ischaemic strokes, we also included transient ischaemic attacks (TIA) in our definition. The latter is seldom captured by comparable post AMI cohorts that show a decline in stroke rates [11, 41]. From 2008, there was national UK guidance that encouraged referral of suspected TIAs to specialist clinics, hence likely improving the identification of TIAs across the country [42]. If this rise in identification was not offset by a corresponding decline in CVA-related hospitalisations, it could help to explain the observed differences. However, there is limited data on TIA incidence trends during the time of our study, meaning our hypothesis is purely speculative.

### Major adverse cardiovascular events

Aside from all-cause mortality, the overall impact of varying trends can be considered through changes in the relative risk of composite MACE. Reassuringly, the risk for MACE at 1- and 5-years decreased through the study period as a consequence of a reduction in mortality and recurrent AMI, although this was temporised by rising rates of HF and stroke. As the latter outcomes have been suggested to fall across other contemporary data [11], it is unsurprising that we observed a smaller magnitude in the change in risk compared to these data. Clinically, the relative change in distributions of our MACE events is important, as it reflects a changing landscape in the complications experienced post AMI in both the shorter (1-year) and longer (5-years) term. Although it does not capture the magnitude of certain complications therein (i.e., degree of HF), it offers insight into how post AMI care should remain focused on secondary CV care for non-mortality outcomes.

### Strengths and limitations

The present study has multiple strengths. Firstly, the use of a linked CPRD dataset allowed us to obtain a substantial sample size throughout the UK, whilst improving the reliability of diagnoses. Unlike other data discussed, our combined use of primary and secondary care data would have captured a wider burden of disease that would have been missed by mere secondary data alone. Furthermore, our data reports on 5-year trends as well as 1-years trends across the primary and secondary outcomes. This gives a descriptive and novel understanding of the real world-effects of longer-term patient outcomes that are not captured from other literature. The study used appropriate statistical methods. We estimated rates using time-to-event models (Cox) which account for censorship more effectively than other regression models (e.g. Poisson), and generated adjusted survival curves and counterfactual analyses, features seldom reported in contemporary literature.

Our study also has limitations. Firstly, the current study findings reflect historic patterns and should therefore be interpreted in this context. Rather than describing current clinical benchmarks, where AMI outcomes and MACE events may have since shifted, it should be interpreted as describing long term-temporal trends in a past healthcare context. Second, our definition of CVA to include ischaemic stroke, intracranial bleeding, and TIA could be considered a limitation. By not stratifying such subcomponents, the clinical significance of the measured outcome may be diluted as the risk and treatment profiles for each are non-similar. Another limitation of the work is the inclusion of only those aged 35 or older—data for this study were obtained as part of a larger HRUK funded project that developed a risk prediction model for recurring AMI in primary care where the inclusion of those under 35 would have been problematic due to very low incidence. Further, as the original project was based for a primary care risk prediction tool, secondary care predictors (such as left ventricular ejection fraction, PCI, ST-elevation MI (STEMI) and non-STEMI) were not extracted into the dataset and thus could not be analysed. Ultimately, we had to work with the data we had available. Electronic health care records may introduce disease misclassification through inaccurate recording, but it has been shown that there is accurate classification of AMI in CRPD [43]. Electronic records may also have missing data, though we attempted to reduce this issue through an imputation model that included all covariates of interest. Finally, and perhaps most obviously, the observational design precludes making definitive causative links between the associations explored within our study, particularly drawn from other literature. However, it should be noted that our cohort would have been invariably subject to many of the explored changes during our study period, such as the increasing use of PCI, due to their national scope with standardised guidelines. As such, they likely contributed to our observations.

## Conclusion

From 2006 to 2014, there was a decrease in the 1- and 5-year risk of all-cause mortality, CV-related mortality, and recurrent AMI across the UK post incident AMI. These observations were consistent across in-hospital and post-discharge outcomes, where changes across in-hospital mortality appeared to have the greatest impact on trends across all-cause mortality. We also observed an increase in risk for incident HF and CVA. These may have been driven by multifactorial factors post-discharge, including survivorship bias and methodological differences.

## Supplementary Information

Supplementary Material 1.

Supplementary Material 2.

## Data Availability

The data for this article are available herein the article and the online supplementary material.
