# Correction: Trends in mortality and major adverse cardiovascular events following incident acute myocardial infarction

**DOI:** 10.1186/s12872-026-06290-x

**Published:** 2026-07-20

**Authors:** Steven Scholfield, Salwa S. Zghebi, Martin K. Rutter, Mamas A. Mamas, Evangelos Kontopantelis

**Affiliations:** 1 Division of Cardiovascular Sciences, School of Medical Sciences, Faculty of Biology, Medicine and Health, University of Manchester, Manchester, UK; 2 Division of Population Health, Health Services Research and Primary Care, School of Health Sciences, Faculty of Biology, Medicine and Health, University of Manchester, Manchester, UK; 3 Division of Diabetes, Endocrinology & Gastroenterology, School of Medical Sciences, Faculty of Biology, Medicine and Health, University of Manchester, Manchester, UK; 4 Keele Cardiovascular Research Group, Centre for Prognosis Research, Institute for Primary Care and Health Sciences, Keele University, Keele, UK; 5 Division of Informatics, Imaging and Data Sciences, School of Health Sciences, Faculty of Biology, Medicine and Health, University of Manchester, Manchester, UK

**Correction: BMC Cardiovasc Disord 26, 125 (2026)**

**https://doi.org/10.1186/s12872-025-05481-2**

In this article [1], the author’s name Martin K Rutter, was incorrectly written as Martin Rutter.

The original article has been corrected.

## References

[1] Scholfield S, Zghebi SS, Rutter M, et al. Trends in mortality and major adverse cardiovascular events following incident acute myocardial infarction. BMC Cardiovasc Disord. 2026;26:125. 10.1186/s12872-025-05481-2. 41514213 10.1186/s12872-025-05481-2 PMC12882593

