# Supplementary material for: Trends in mortality and major adverse cardiovascular events following incident acute myocardial infarction
Source: BMC Cardiovasc Disord. 2026 Jan 10;26:125. doi: 10.1186/s12872-025-05481-2 (PMC12882593; doi:10.1186/s12872-025-05481-2)
Supplement: Supplementary file 1 — Supplementary Material 1. [file 12872_2025_5481_MOESM1_ESM.docx]

**Supplementary Materials 1**

Trends in Mortality and Major Adverse Cardiovascular Events Following Incident Acute Myocardial Infarction

**Corresponding author:**

Dr Steven Scholfield

Division of Cardiovascular Sciences

University of Manchester

United Kingdom

Email. [steven.scholfield@postgrad.manchester.ac.uk](mailto:steven.scholfield@postgrad.manchester.ac.uk)

**Table S1**: Diagnostic Codes for Incident Event and Outcomes

| **Incident event** | **Codes** |
| --- | --- |
| Acute myocardial infarction (AMI) | SNOMED codes |
| **Outcomes** |  |
| Cardiovascular-related death | ICD-10: I20-I25, I30-52, I60-I99, G45, G46 |
| Heart failure | SNOMED codes *and* ICD-10: I11.0, I13.0, I13.2, I50.0, I50.1, I50.9 |
| Recurrent AMI | Identical SNOMED codes for AMI on any date after the incident date |
| Cerebrovascular accidents | SNOMED codes *and* ICD-10: I60-I69 |

**Table S2**: Hazard Ratios for All-Cause Mortality at 1-Year in Comprehensive Cox Analysis with the Influence of Pre-defined Variables Highlighted

| **Outcomes** | **Hazard Ratio** | **P-Value** | **Lower limit 95% CI** | **Upper limit 95% CI** |
| --- | --- | --- | --- | --- |
| 2007 | 0.911 | 0.034 | 0.836 | 0.993 |
| 2008 | 0.880 | 0.003 | 0.808 | 0.958 |
| 2009 | 0.894 | 0.010 | 0.820 | 0.974 |
| 2010 | 0.875 | 0.002 | 0.803 | 0.954 |
| 2011 | 0.887 | 0.006 | 0.815 | 0.966 |
| 2012 | 0.830 | <0.001 | 0.762 | 0.905 |
| 2013 | 0.799 | <0.001 | 0.732 | 0.872 |
| 2014 | 0.820 | <0.001 | 0.751 | 0.895 |
| Age (continuous variable) | 1.067 | <0.001 | 1.065 | 1.070 |
| Female | 0.935 | 0.002 | 0.896 | 0.976 |
| Body mass index (continuous variable) | 0.971 | <0.001 | 0.966 | 0.975 |
| Coronary Heart Disease | 0.821 | <0.001 | 0.783 | 0.862 |
| Ethnicity (non-White) | 0.840 | 0.001 | 0.759 | 0.929 |
| IMD Q2 | 1.076 | 0.025 | 1.009 | 1.147 |
| IMD Q3 | 1.124 | <0.001 | 1.053 | 1.199 |
| IMD Q4 | 1.198 | <0.001 | 1.123 | 1.279 |
| IMD Q5-most deprived | 1.263 | <0.001 | 1.183 | 1.349 |
| Anticoagulants | 1.273 | <0.001 | 1.196 | 1.354 |
| Antihypertensives | 1.397 | <0.001 | 1.302 | 1.500 |
| Antiplatelets | 1.230 | <0.001 | 1.167 | 1.296 |
| Lipid lowering therapy | 0.902 | <0.001 | 0.856 | 0.951 |
| Smoking status | 0.950 | 0.001 | 0.920 | 0.980 |
| Diabetes | 1.427 | <0.001 | 1.357 | 1.501 |
| Cerebrovascular disease | 1.269 | <0.001 | 1.203 | 1.339 |
| Heart failure | 1.618 | <0.001 | 1.527 | 1.713 |
| Hyperlipidaemia | 0.953 | 0.080 | 0.902 | 1.006 |
| Chronic pulmonary disease | 1.394 | <0.001 | 1.325 | 1.467 |
| Chronic kidney disease | 1.273 | <0.001 | 1.216 | 1.332 |
| Cox Regression Analysis for All-cause mortality at 1-year with the influence of each variable on the hazard ratios (HRs) shown. Pre-defined variables for analysis are highlighted. For sex, ethnicity, and IMD status, the references were male, White, and IMD Q1 respectively. CI: confidence interval. IMD: Index of Multiple Deprivation. Q: Quintile. Statistical significance at P<0.05. | | | | |

**Table S3**: Hazard Ratios for All-Cause Mortality at 5-Year in Comprehensive Cox Analysis with the Influence of Pre-defined Variables Highlighted

| **Outcomes** | **Hazard Ratio** | **P-Value** | **Lower limit 95% CI** | **Upper limit 95% CI** |
| --- | --- | --- | --- | --- |
| 2007 | 0.954 | 0.087 | 0.904 | 1.007 |
| 2008 | 0.925 | 0.004 | 0.877 | 0.976 |
| 2009 | 0.928 | 0.006 | 0.879 | 0.979 |
| 2010 | 0.886 | <0.001 | 0.840 | 0.935 |
| 2011 | 0.905 | <0.001 | 0.858 | 0.954 |
| 2012 | 0.857 | <0.001 | 0.812 | 0.904 |
| 2013 | 0.830 | <0.001 | 0.786 | 0.876 |
| 2014 | 0.824 | <0.001 | 0.780 | 0.871 |
| Age (continuous variable) | 1.076 | <0.001 | 1.075 | 1.078 |
| Female | 0.948 | <0.001 | 0.923 | 0.973 |
| Body mass index (continuous variable) | 0.978 | <0.001 | 0.976 | 0.981 |
| Coronary heart disease | 0.885 | <0.001 | 0.859 | 0.911 |
| Ethnicity (non-White) | 1.063 | 0.019 | 1.010 | 1.118 |
| IMD Q2 | 1.068 | 0.001 | 1.026 | 1.112 |
| IMD Q3 | 1.131 | <0.001 | 1.087 | 1.178 |
| IMD Q4 | 1.208 | <0.001 | 1.160 | 1.258 |
| IMD Q5-most deprived | 1.297 | <0.001 | 1.245 | 1.351 |
| Anticoagulants | 1.274 | <0.001 | 1.225 | 1.326 |
| Antihypertensives | 1.344 | <0.001 | 1.288 | 1.402 |
| Antiplatelet | 1.261 | <0.001 | 1.220 | 1.303 |
| Lipid lowering therapy | 0.921 | <0.001 | 0.891 | 0.952 |
| Smoking status | 0.882 | <0.001 | 0.865 | 0.899 |
| Diabetes | 1.474 | <0.001 | 1.428 | 1.521 |
| Cerebrovascular disease | 1.277 | <0.001 | 1.234 | 1.322 |
| Heart failure | 1.568 | <0.001 | 1.510 | 1.629 |
| Hyperlipidaemia | 0.947 | 0.001 | 0.917 | 0.979 |
| Chronic pulmonary disease | 1.481 | <0.001 | 1.435 | 1.528 |
| Chronic kidney disease | 1.266 | <0.001 | 1.230 | 1.303 |
| Cox Regression Analysis for All-cause mortality at 5-year with the influence of each variable on the hazard ratios (HRs) shown. Pre-defined variables for analysis are highlighted. For sex, ethnicity, and IMD status, the references were male, White, and IMD Q1 respectively. CI: confidence interval. IMD: Index of Multiple Deprivation. Q: Quintile. Statistical significance at P<0.05. | | | | |

**Table S4**: All-Cause Mortality at 1-Year After Discharge (Extracted from Full Cox Model – Only the Hazard Ratios for Incident Years Shown)

| **Incident Year** | **Hazard Ratio** | **P-Value** | **Lower limit 95% CI** | **Upper limit 95% CI** |
| --- | --- | --- | --- | --- |
| 2007 | 0.907 | 0.032 | 0.830 | 0.992 |
| 2008 | 0.895 | 0.014 | 0.820 | 0.977 |
| 2009 | 0.894 | 0.014 | 0.818 | 0.977 |
| 2010 | 0.874 | 0.003 | 0.800 | 0.955 |
| 2011 | 0.899 | 0.017 | 0.824 | 0.981 |
| 2012 | 0.832 | <0.001 | 0.762 | 0.909 |
| 2013 | 0.804 | <0.001 | 0.734 | 0.880 |
| 2014 | 0.825 | <0.001 | 0.754 | 0.903 |
| Cox regression for All-Cause Mortality at 1-Year from the date of discharge. Reference incident year is 2006. | | | | |

**Table S5**: CV-Related Mortality at 1-Year After Discharge (Extracted from Full Cox Model – Only the Hazard Ratios for Incident Years Shown)

| **Incident Year** | **Hazard Ratio** | **P-Value** | **Lower limit 95% CI** | **Upper limit 95% CI** |
| --- | --- | --- | --- | --- |
| 2007 | 0.812 | 0.002 | 0.711 | 0.928 |
| 2008 | 0.843 | 0.009 | 0.740 | 0.959 |
| 2009 | 0.820 | 0.003 | 0.719 | 0.935 |
| 2010 | 0.733 | <0.001 | 0.641 | 0.838 |
| 2011 | 0.688 | <0.001 | 0.601 | 0.788 |
| 2012 | 0.662 | <0.001 | 0.578 | 0.759 |
| 2013 | 0.622 | <0.001 | 0.541 | 0.715 |
| 2014 | 0.690 | <0.001 | 0.602 | 0.791 |
| Cox regression for CV-related Mortality at 1-Year from the date of discharge. Reference incident year is 2006. | | | | |

**Table S6**: Incident Heart Failure at 1-Year After Discharge (Extracted from Full Cox Model – Only the Hazard Ratios for Incident Years Shown)

| **Incident Year** | **Hazard Ratio** | **P-Value** | **Lower limit 95% CI** | **Upper limit 95% CI** |
| --- | --- | --- | --- | --- |
| 2007 | 0.937 | 0.194 | 0.850 | 1.033 |
| 2008 | 0.991 | 0.853 | 0.901 | 1.090 |
| 2009 | 0.976 | 0.617 | 0.886 | 1.074 |
| 2010 | 0.983 | 0.720 | 0.893 | 1.081 |
| 2011 | 1.012 | 0.805 | 0.921 | 1.112 |
| 2012 | 1.077 | 0.118 | 0.981 | 1.182 |
| 2013 | 1.110 | 0.028 | 1.011 | 1.218 |
| 2014 | 1.194 | <0.001 | 1.089 | 1.309 |
| Cox regression for Incident Heart Failure at 1-Year from the date of discharge. Reference incident year is 2006. | | | | |

**Table S7**: Recurrent Acute Myocardial Infarction at 1-Year After Discharge (Extracted from Full Cox Model – Only the Hazard Ratios for Incident Years Shown)

| **Incident Year** | **Hazard Ratio** | **P-Value** | **Lower limit 95% CI** | **Upper limit 95% CI** |
| --- | --- | --- | --- | --- |
| 2007 | 0.985 | 0.830 | 0.857 | 1.132 |
| 2008 | 0.928 | 0.292 | 0.807 | 1.067 |
| 2009 | 0.870 | 0.056 | 0.755 | 1.003 |
| 2010 | 0.703 | <0.001 | 0.605 | 0.816 |
| 2011 | 0.701 | <0.001 | 0.605 | 0.812 |
| 2012 | 0.732 | <0.001 | 0.632 | 0.846 |
| 2013 | 0.662 | <0.001 | 0.570 | 0.770 |
| 2014 | 0.803 | 0.003 | 0.695 | 0.928 |
| Cox regression for Recurrent Acute Myocardial Infarction at 1-Year from the date of discharge. Reference incident year is 2006. | | | | |

**Table S8**: Cerebrovascular Accidents at 1-Year After Discharge (Extracted from Full Cox Model – Only the Hazard Ratios for Incident Years Shown)

| **Incident Year** | **Hazard Ratio** | **P-Value** | **Lower limit 95% CI** | **Upper limit 95% CI** |
| --- | --- | --- | --- | --- |
| 2007 | 0.936 | 0.337 | 0.817 | 1.072 |
| 2008 | 1.019 | 0.779 | 0.894 | 1.162 |
| 2009 | 1.043 | 0.534 | 0.914 | 1.189 |
| 2010 | 1.190 | 0.007 | 1.048 | 1.350 |
| 2011 | 1.112 | 0.102 | 0.979 | 1.263 |
| 2012 | 1.167 | 0.016 | 1.029 | 1.324 |
| 2013 | 1.084 | 0.223 | 0.952 | 1.233 |
| 2014 | 1.110 | 0.115 | 0.975 | 1.263 |
| Cox regression for Cerebrovascular accidents at 1-Year from the date of discharge. Reference incident year is 2006. | | | | |

**Table S9**: Major Adverse Cardiovascular Events at 1-Year After Discharge (Extracted from Full Cox Model – Only the Hazard Ratios for Incident Years Shown)

| **Incident Year** | **Hazard Ratio** | **P-Value** | **Lower limit 95% CI** | **Upper limit 95% CI** |
| --- | --- | --- | --- | --- |
| 2007 | 0.930 | 0.054 | 0.864 | 1.001 |
| 2008 | 0.982 | 0.614 | 0.913 | 1.055 |
| 2009 | 0.962 | 0.295 | 0.894 | 1.035 |
| 2010 | 0.935 | 0.071 | 0.869 | 1.006 |
| 2011 | 0.939 | 0.088 | 0.874 | 1.009 |
| 2012 | 0.971 | 0.419 | 0.904 | 1.043 |
| 2013 | 0.960 | 0.264 | 0.893 | 1.032 |
| 2014 | 1.040 | 0.278 | 0.969 | 1.117 |
| Cox regression for Major Adverse Cardiovascular Events at 1-Year from the date of discharge. Reference incident year is 2006. | | | | |

**Table S10**: All-Cause Mortality up to 5-Years After Discharge (Extracted from Full Cox Model – Only the Hazard Ratios for Incident Years Shown)

| **Incident Year** | **Hazard Ratio** | **P-Value** | **Lower limit 95% CI** | **Upper limit 95% CI** |
| --- | --- | --- | --- | --- |
| 2007 | 0.950 | 0.067 | 0.900 | 1.004 |
| 2008 | 0.930 | 0.008 | 0.881 | 0.981 |
| 2009 | 0.929 | 0.007 | 0.880 | 0.980 |
| 2010 | 0.887 | <0.001 | 0.840 | 0.937 |
| 2011 | 0.908 | <0.001 | 0.861 | 0.958 |
| 2012 | 0.862 | <0.001 | 0.817 | 0.910 |
| 2013 | 0.833 | <0.001 | 0.788 | 0.880 |
| 2014 | 0.826 | <0.001 | 0.782 | 0.874 |
| Cox regression for All-Cause Mortality up to 5-Years from the date of discharge. Reference incident year is 2006. | | | | |

**Table S11**: Cardiovascular-Related Mortality up to 5-Years After Discharge (Extracted from Full Cox Model – Only the Hazard Ratios for Incident Years Shown)

| **Incident Year** | **Hazard Ratio** | **P-Value** | **Lower limit 95% CI** | **Upper limit 95% CI** |
| --- | --- | --- | --- | --- |
| 2007 | 0.907 | 0.027 | 0.832 | 0.989 |
| 2008 | 0.891 | 0.008 | 0.818 | 0.971 |
| 2009 | 0.822 | <0.001 | 0.753 | 0.897 |
| 2010 | 0.785 | <0.001 | 0.719 | 0.857 |
| 2011 | 0.742 | <0.001 | 0.679 | 0.811 |
| 2012 | 0.700 | <0.001 | 0.640 | 0.766 |
| 2013 | 0.663 | <0.001 | 0.605 | 0.727 |
| 2014 | 0.684 | <0.001 | 0.624 | 0.749 |
| Cox regression for Cardiovascular-Related Mortality up to 5-Years from the date of discharge. Reference incident year is 2006. | | | | |

**Table S12**: Incident Heart Failure up to 5-Years After Discharge (Extracted from Full Cox Model – Only the Hazard Ratios for Incident Years Shown)

| **Incident Year** | **Hazard Ratio** | **P-Value** | **Lower limit 95% CI** | **Upper limit 95% CI** |
| --- | --- | --- | --- | --- |
| 2007 | 0.932 | 0.058 | 0.866 | 1.002 |
| 2008 | 0.991 | 0.808 | 0.923 | 1.064 |
| 2009 | 0.983 | 0.631 | 0.914 | 1.056 |
| 2010 | 0.999 | 0.969 | 0.930 | 1.072 |
| 2011 | 1.003 | 0.941 | 0.934 | 1.076 |
| 2012 | 1.033 | 0.366 | 0.963 | 1.107 |
| 2013 | 1.106 | 0.005 | 1.032 | 1.185 |
| 2014 | 1.158 | <0.001 | 1.081 | 1.241 |
| Cox regression for Incident Heart Failure up to 5-Years from the date of discharge. Reference incident year is 2006. | | | | |

**Table S13**: Recurrent Acute Myocardial Infarction up to 5-Years After Discharge (Extracted from Full Cox Model – Only the Hazard Ratios for Incident Years Shown)

| **Incident Year** | **Hazard Ratio** | **P-Value** | **Lower limit 95% CI** | **Upper limit 95% CI** |
| --- | --- | --- | --- | --- |
| 2007 | 0.919 | 0.115 | 0.828 | 1.021 |
| 2008 | 0.903 | 0.053 | 0.814 | 1.001 |
| 2009 | 0.877 | 0.014 | 0.789 | 0.973 |
| 2010 | 0.749 | <0.001 | 0.673 | 0.835 |
| 2011 | 0.745 | <0.001 | 0.669 | 0.829 |
| 2012 | 0.688 | <0.001 | 0.617 | 0.767 |
| 2013 | 0.686 | <0.001 | 0.614 | 0.766 |
| 2014 | 0.750 | <0.001 | 0.673 | 0.836 |
| Cox regression for Recurrent Acute Myocardial Infarction up to 5-Years from the date of discharge. Reference incident year is 2006. | | | | |

**Table S14**: Cerebrovascular Accidents up to 5-Years After Discharge (Extracted from Full Cox Model – Only the Hazard Ratios for Incident Years Shown)

| **Incident Year** | **Hazard Ratio** | **P-Value** | **Lower limit 95% CI** | **Upper limit 95% CI** |
| --- | --- | --- | --- | --- |
| 2007 | 1.030 | 0.508 | 0.944 | 1.122 |
| 2008 | 1.013 | 0.759 | 0.931 | 1.104 |
| 2009 | 1.051 | 0.252 | 0.965 | 1.145 |
| 2010 | 1.117 | 0.010 | 1.027 | 1.214 |
| 2011 | 1.099 | 0.027 | 1.011 | 1.195 |
| 2012 | 1.115 | 0.010 | 1.027 | 1.212 |
| 2013 | 1.082 | 0.065 | 0.995 | 1.177 |
| 2014 | 1.153 | 0.001 | 1.060 | 1.254 |
| Cox regression for Cerebrovascular Accidents up to 5-Years from the date of discharge. Reference incident year is 2006. | | | | |

**Table S15**: Major Adverse Cardiovascular Events up to 5-Years After Discharge (Extracted from Full Cox Model – Only the Hazard Ratios for Incident Years Shown)

| **Incident Year** | **Hazard Ratio** | **P-Value** | **Lower limit 95% CI** | **Upper limit 95% CI** |
| --- | --- | --- | --- | --- |
| 2007 | 0.951 | 0.070 | 0.901 | 1.004 |
| 2008 | 0.967 | 0.214 | 0.916 | 1.020 |
| 2009 | 0.968 | 0.245 | 0.918 | 1.022 |
| 2010 | 0.953 | 0.080 | 0.903 | 1.006 |
| 2011 | 0.964 | 0.172 | 0.914 | 1.016 |
| 2012 | 0.950 | 0.058 | 0.900 | 1.002 |
| 2013 | 0.967 | 0.214 | 0.916 | 1.020 |
| 2014 | 1.022 | 0.415 | 0.969 | 1.079 |
| Cox regression for Major Adverse Cardiovascular Events up to 5-Years from the date of discharge. Reference incident year is 2006. | | | | |

**Table S16**: Odds of Death Prior to Discharge (In-Hospital Death) Compared to the Reference Year (Extracted from Full Model – Only the Odds Ratios for Incident Years Shown)

| **Incident Year** | **Odds Ratio** | **P-Value** | **Lower limit 95% CI** | **Upper limit 95% CI** |
| --- | --- | --- | --- | --- |
| 2007 | 0.832 | 0.020 | 0.712 | 0.972 |
| 2008 | 0.733 | <0.001 | 0.626 | 0.859 |
| 2009 | 0.683 | <0.001 | 0.581 | 0.803 |
| 2010 | 0.578 | <0.001 | 0.489 | 0.684 |
| 2011 | 0.542 | <0.001 | 0.457 | 0.643 |
| 2012 | 0.434 | <0.001 | 0.362 | 0.521 |
| 2013 | 0.384 | <0.001 | 0.318 | 0.465 |
| 2014 | 0.360 | <0.001 | 0.296 | 0.438 |
| Logistic regression for odds of in-hospital death compared to the reference year. Reference incident year is 2006. | | | | |

**Table S17**: Odds of Cardiovascular-Related Death Prior to Discharge Compared to the Reference Year (Extracted from Full Model – Only the Odds Ratios for Incident Years Shown)

| **Incident Year** | **Odds Ratio** | **P-Value** | **Lower limit 95% CI** | **Upper limit 95% CI** |
| --- | --- | --- | --- | --- |
| 2007 | 0.857 | 0.231 | 0.665 | 1.103 |
| 2008 | 0.712 | 0.011 | 0.548 | 0.924 |
| 2009 | 0.679 | 0.004 | 0.520 | 0.886 |
| 2010 | 0.472 | <0.001 | 0.352 | 0.633 |
| 2011 | 0.462 | <0.001 | 0.345 | 0.619 |
| 2012 | 0.433 | <0.001 | 0.322 | 0.583 |
| 2013 | 0.336 | <0.001 | 0.243 | 0.465 |
| 2014 | 0.396 | <0.001 | 0.291 | 0.540 |
| Logistic regression for odds of in-hospital Cardiovascular-Related Death compared to the reference year. Reference incident year is 2006. | | | | |

**Table S18**: Odds of Incident Heart Failure Prior to Discharge Compared to the Reference Year (Extracted from Full Model – Only the Odds Ratios for Incident Years Shown)

| **Incident Year** | **Odds Ratio** | **P-Value** | **Lower limit 95% CI** | **Upper limit 95% CI** |
| --- | --- | --- | --- | --- |
| 2007 | 0.954 | 0.260 | 0.878 | 1.036 |
| 2008 | 0.932 | 0.092 | 0.859 | 1.012 |
| 2009 | 0.957 | 0.293 | 0.882 | 1.039 |
| 2010 | 0.869 | 0.001 | 0.800 | 0.944 |
| 2011 | 0.834 | <0.001 | 0.768 | 0.906 |
| 2012 | 0.786 | <0.001 | 0.723 | 0.845 |
| 2013 | 0.814 | <0.001 | 0.749 | 0.885 |
| 2014 | 0.699 | <0.001 | 0.642 | 0.762 |
| Logistic regression for odds of in-hospital Incident Heart Failure compared to the reference year. Reference incident year is 2006. | | | | |

**Table S19**: Odds of Recurrent Acute Myocardial Infarction Prior to Discharge Compared to the Reference Year (Extracted from Full Model – Only the Odds Ratios for Incident Years Shown)

| **Incident Year** | **Odds Ratio** | **P-Value** | **Lower limit 95% CI** | **Upper limit 95% CI** |
| --- | --- | --- | --- | --- |
| 2007 | 0.959 | 0.385 | 0.874 | 1.053 |
| 2008 | 0.920 | 0.080 | 0.838 | 1.010 |
| 2009 | 0.850 | 0.001 | 0.773 | 0.935 |
| 2010 | 0.636 | <0.001 | 0.575 | 0.704 |
| 2011 | 0.544 | <0.001 | 0.490 | 0.604 |
| 2012 | 0.538 | <0.001 | 0.485 | 0.597 |
| 2013 | 0.520 | <0.001 | 0.467 | 0.578 |
| 2014 | 0.452 | <0.001 | 0.405 | 0.505 |
| Logistic regression for odds of in-hospital recurrent Acute Myocardial Infarction compared to the reference year of 2006. | | | | |

**Table S20**: Odds of Cerebrovascular Accidents Prior to Discharge Compared to the Reference Year (Extracted from Full Model – Only the Odds Ratios for Incident Years Shown)

| **Incident Year** | **Odds Ratio** | **P-Value** | **Lower limit 95% CI** | **Upper limit 95% CI** |
| --- | --- | --- | --- | --- |
| 2007 | 0.935 | 0.219 | 0.839 | 1.041 |
| 2008 | 0.848 | 0.003 | 0.761 | 0.946 |
| 2009 | 0.843 | 0.002 | 0.755 | 0.940 |
| 2010 | 0.838 | 0.001 | 0.751 | 0.934 |
| 2011 | 0.758 | <0.001 | 0.679 | 0.847 |
| 2012 | 0.661 | <0.001 | 0.590 | 0.740 |
| 2013 | 0.604 | <0.001 | 0.538 | 0.678 |
| 2014 | 0.558 | <0.001 | 0.495 | 0.629 |
| Logistic regression for odds of in-hospital Cerebrovascular Accidents compared to the reference year. Reference incident year is 2006. | | | | |

**Table S21**: Odds of Major Adverse Cardiovascular Events Prior to Discharge Compared to the Reference Year (Extracted from Full Model – Only the Odds Ratios for Incident Years Shown)

| **Incident Year** | **Odds Ratio** | **P-Value** | **Lower limit 95% CI** | **Upper limit 95% CI** |
| --- | --- | --- | --- | --- |
| 2007 | 0.956 | 0.165 | 0.897 | 1.019 |
| 2008 | 0.896 | 0.001 | 0.841 | 0.954 |
| 2009 | 0.863 | <0.001 | 0.810 | 0.920 |
| 2010 | 0.796 | <0.001 | 0.746 | 0.849 |
| 2011 | 0.706 | <0.001 | 0.661 | 0.753 |
| 2012 | 0.694 | <0.001 | 0.650 | 0.740 |
| 2013 | 0.643 | <0.001 | 0.602 | 0.689 |
| 2014 | 0.572 | <0.001 | 0.535 | 0.612 |
| Logistic regression for odds of in-hospital Major Adverse Cardiovascular Events compared to the reference year of 2006. | | | | |

**Counterfactual Decomposition Analysis of Mortality**

We observed the in-hospital and post-discharge trends in the odds and hazard for mortality decrease in 2014 compared to 2006, respectively. Although these values are not directly comparable, they are indicative that inpatient and post-discharge care improved, suggesting both phases of care contributed to the trends we observed in all-cause mortality. However, they do not quantify the contribution of each phase on the falling trends and hence do not indicate if proxy improvements in inpatient care contributed more (or less) than improvements in post-discharge care.

To explore the relative contributions, we performed a counterfactual decomposition analysis comparing 2014 to 2006. This technique divides the total change in mortality into segments (inpatient- and post-discharge mortality) (Table S22), and models changes in mortality under counterfactual conditions for each segment.

**Table S22**: In-hospital, post-discharge and total mortality rates in 2014.

| **Incident Year** | **In-hospital mortality** | **Post-discharge mortality** | **Total mortality** |
| --- | --- | --- | --- |
| 2014 | 1.4% | 5.9% | 7.2% |
| **Table S22.** Table of the in-hospital, post-discharge, and total mortality rates in 2014. | | | |

Subsequently, the difference between total mortality and each counterfactual mortality is the contribution of that segment to changes in 1-year mortality. For our study, we modelled two conditions: 1) the expected 1-year mortality rate (in 2014) if in-hospital mortality maintained its baseline rate (in 2006), and 2) the expected 1-year mortality rate (in 2014) if post-discharge mortality maintained its baseline rate (in 2006) (Table S23).

**Table S23**: Counterfactual decomposition analysis of total and component mortalities comparing 2014 to 2006

| **Incident Year** | **Counter-factual in-hospital mortality** | **Counter-factual post-discharge mortality** | **Absolute contribution of ∆in-hospital mortality to ∆total mortality** | **Absolute contribution of ∆post-discharge mortality to ∆total mortality** | **Relative contribution of ∆in-hospital mortality to ∆total mortality** | **Relative contribution of ∆post-discharge mortality to ∆total mortality** |
| --- | --- | --- | --- | --- | --- | --- |
| 2014 | 12% | 9.0% | -4.3% | -1.7% | 72% | 28% |
| **Table S23.** Stata v17 output of the in-hospital, post-discharge, and total mortality in 2014 counterfactual decomposition analysis. Total mortality was divided into in-hospital- and post-discharge mortality, comparing 2014 to the reference year 2006. Counterfactual mortality estimates were created for each of in-patient and post-discharge components. Decimal values represent proportions: multiplying by 100 will express these values as percentages. The change in total mortality in 2014, compared to 2006, is the sum of the last two columns. | | | | | | |

The total mortality at 1-year for 2014 was 7.2%. The counterfactual in-hospital mortality was 11.5%, whilst the counterfactual post-discharge mortality was 9.0%. Therefore, the inpatient contribution to 1-year mortality in 2014 compared to 2006 was -4.3% (total mortality minus counterfactual inpatient mortality), and the post-discharge contribution was -1.7%. As a proportion of the total combined change of -6.0%, this analysis suggests that 72% of the reduction was due to improvements in in-hospital survival, whilst the remaining 28% was due to improved post-discharge outcomes (Table S23 - highlighted).

**Competing Risk Analysis – 1-year outcomes**

To assess for competing risk of all-cause mortality on the secondary outcomes, a Fine and Gray competing risk regression model was developed in Stata v17 with the stcrreg function. The outputs for 1-year and 5-year outcomes across CV-related mortality, incident HF, CVA, and recurrent AMI with the sub-distribution hazard ratios shown across each variable.

**Table S24:** Competing Risk Analysis for CV-related mortality at 1-year post AMI

| 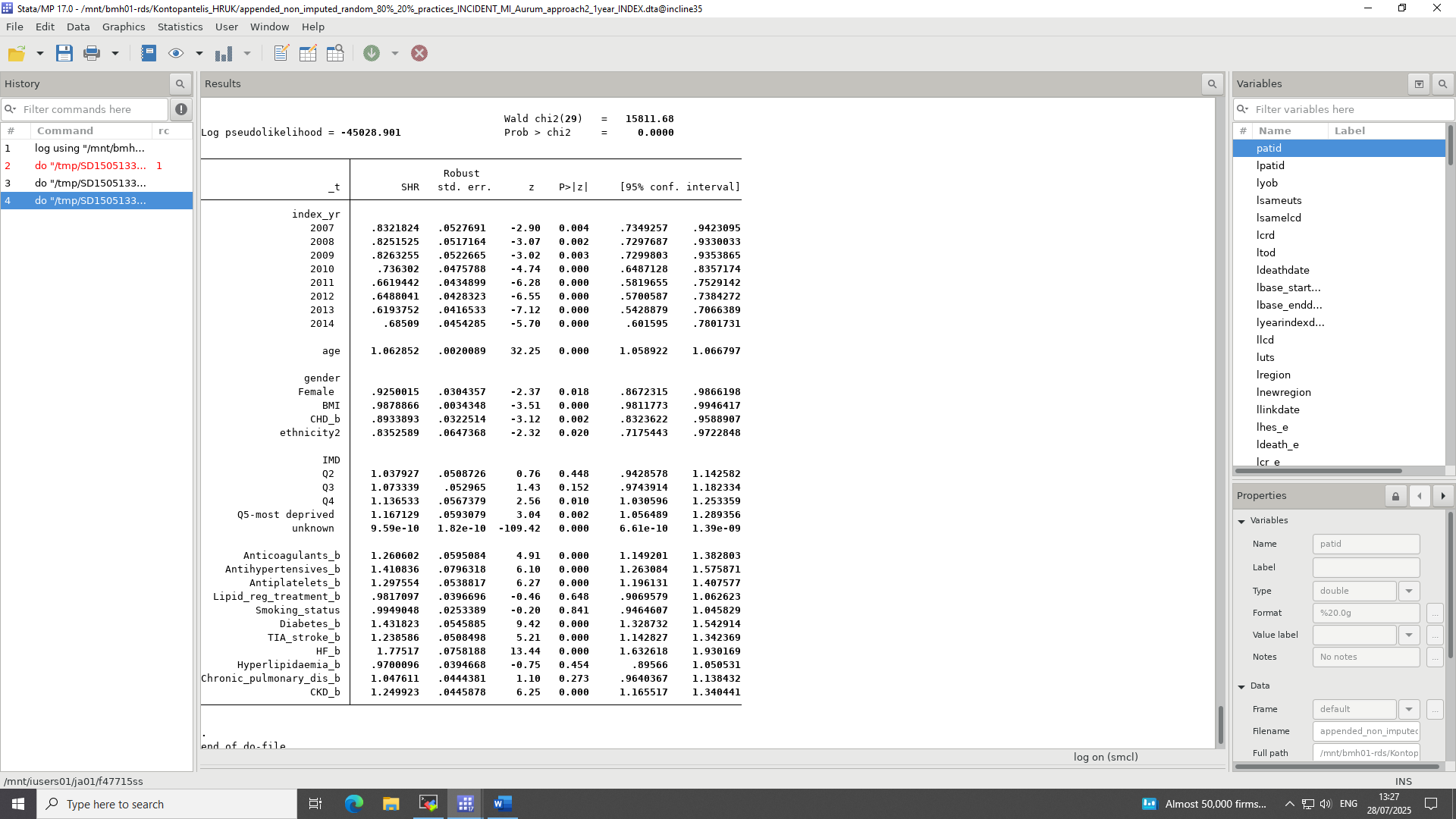 |
| --- |
| Competing Risks Analysis for CV-related mortality at 1-year with the influence of each variable shown. The competing risk was all-cause mortality with non-CV deaths. For sex, ethnicity, and IMD status, the references were male, White, and IMD Q1 respectively. CI: confidence interval. IMD: Index of Multiple Deprivation. SHR: Sub-distribution hazard ratio. Q: Quintile. Statistical significance at P<0.05. |

**Table S25:** Competing Risk Analysis for incident HF at 1-year post AMI

| 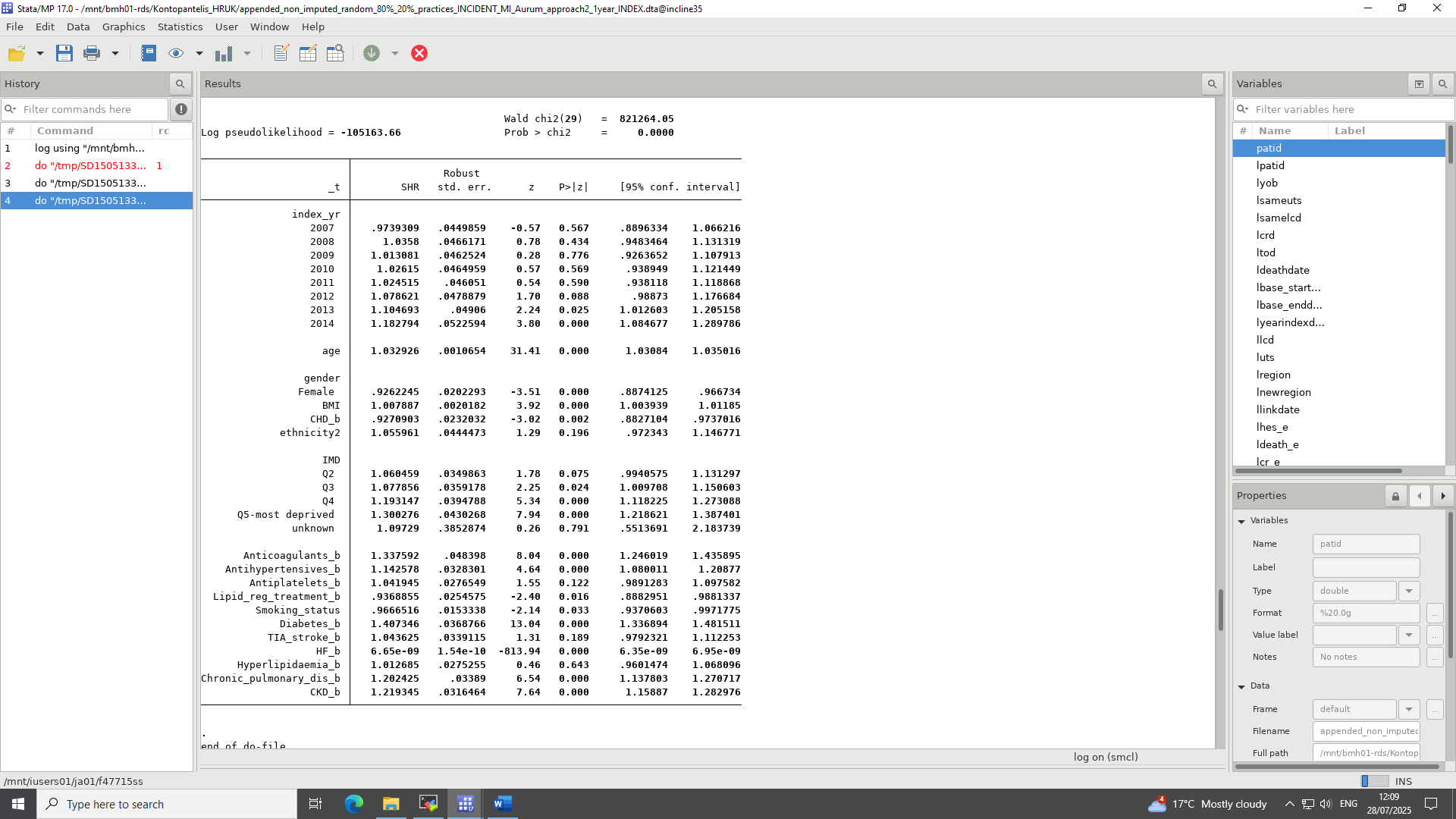 |
| --- |
| Competing Risks Analysis for incident HF at 1-year with the influence of each variable shown. The competing risk was all-cause mortality. For sex, ethnicity, and IMD status, the references were male, White, and IMD Q1 respectively. CI: confidence interval. IMD: Index of Multiple Deprivation. SHR: Sub-distribution hazard ratio. Q: Quintile. Statistical significance at P<0.05. |

**Table S26:** Competing Risk Analysis for CVA at 1-year post AMI

| 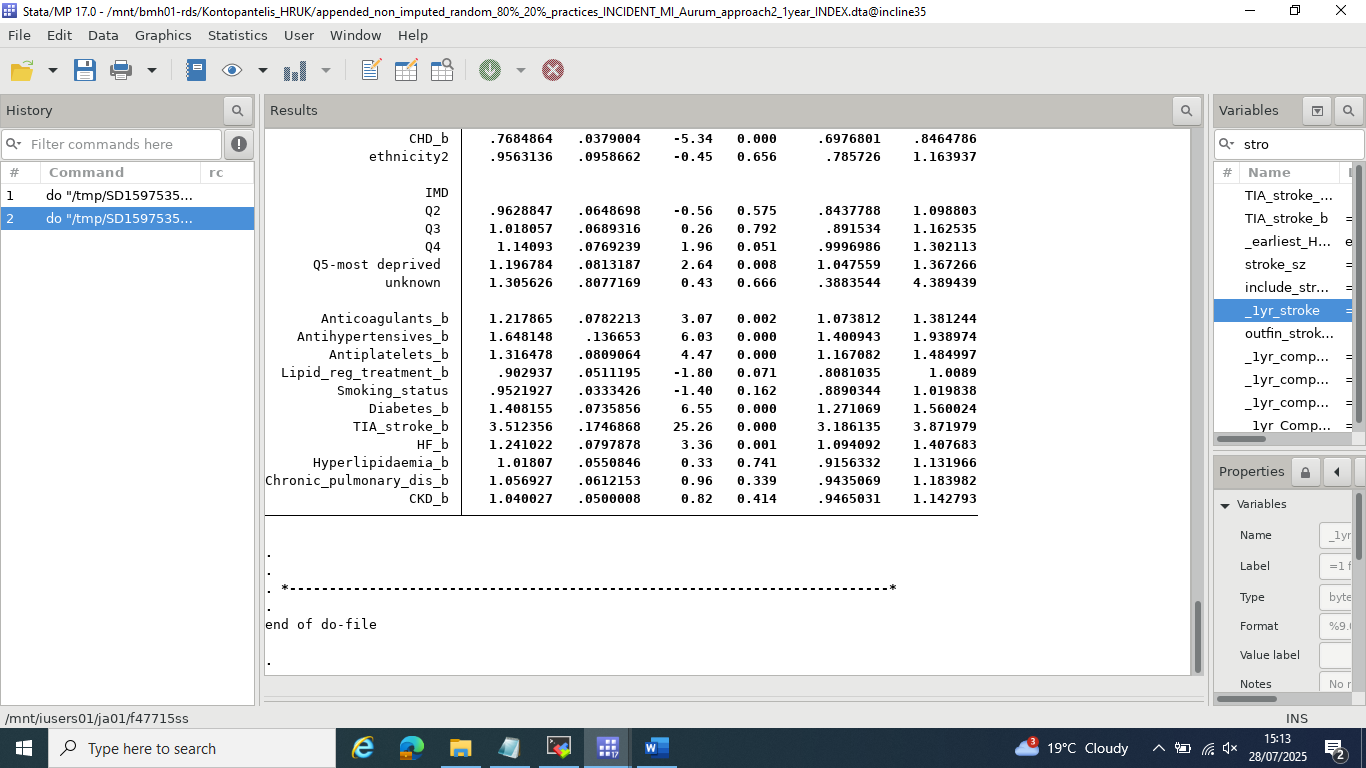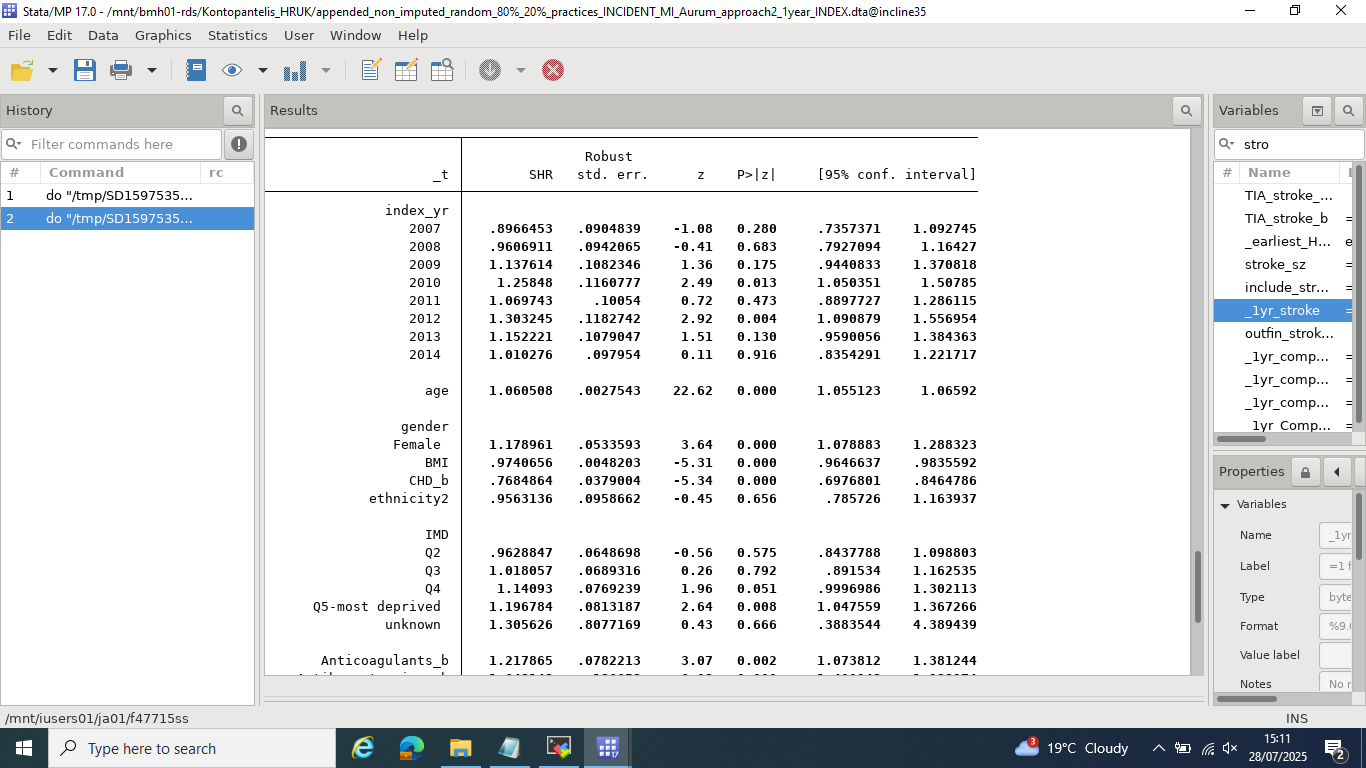 |
| --- |
| Competing Risks Analysis for CVA at 1-year with the influence of each variable shown. The competing risk was all-cause mortality. For sex, ethnicity, and IMD status, the references were male, White, and IMD Q1 respectively. CI: confidence interval. IMD: Index of Multiple Deprivation. SHR: Sub-distribution hazard ratio. Q: Quintile. Statistical significance at P<0.05. |

**Table S27:** Competing Risk Analysis for recurrent AMI at 1-year post AMI

| 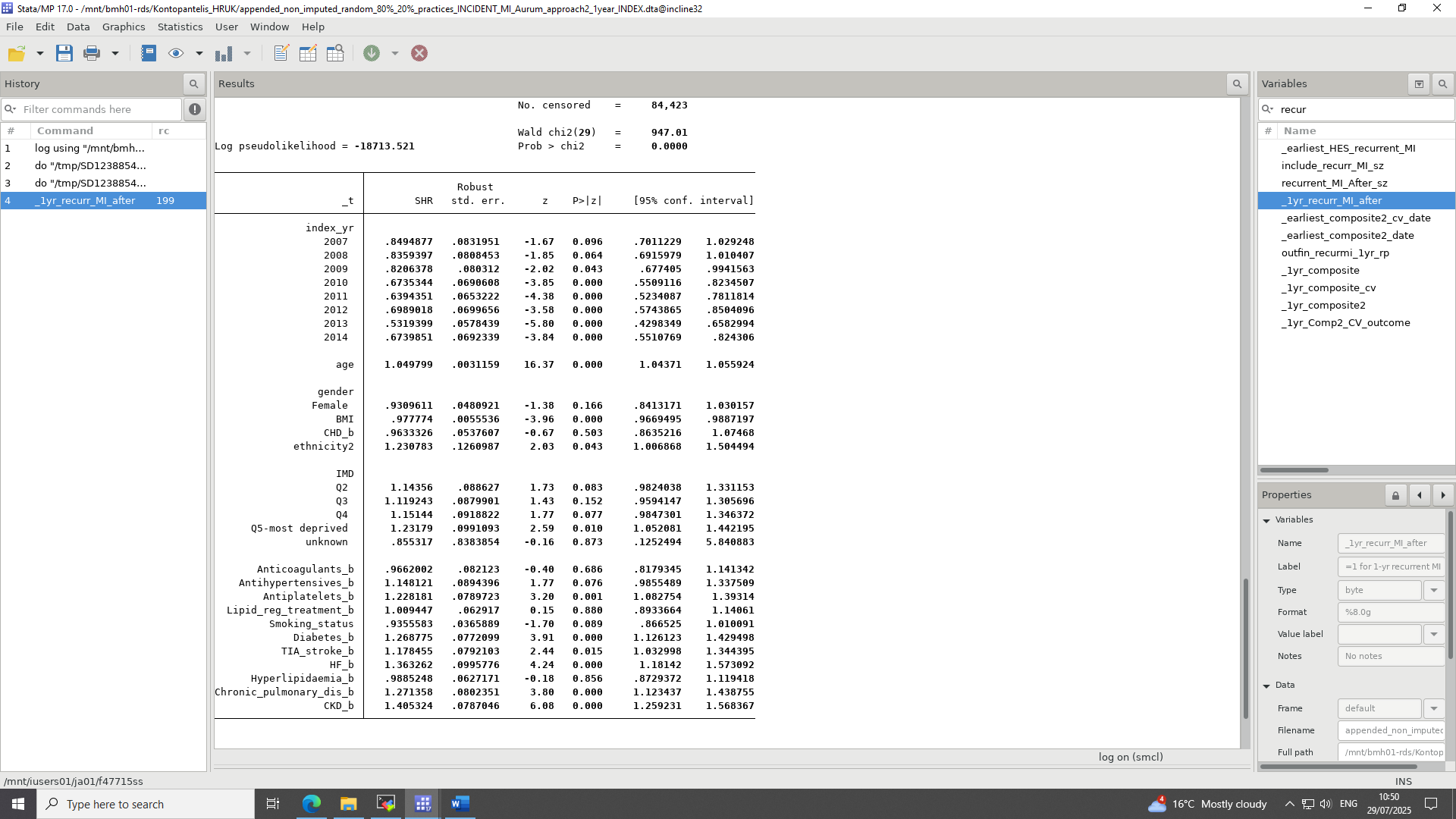 |
| --- |
| Competing Risks Analysis for recurrent AMI at 1-year with the influence of each variable shown. The competing risk was all-cause mortality. For sex, ethnicity, and IMD status, the references were male, White, and IMD Q1 respectively. CI: confidence interval. IMD: Index of Multiple Deprivation. SHR: Sub-distribution hazard ratio. Q: Quintile. Statistical significance at P<0.05. |

**Competing Risk Analysis – 5-year outcomes**

**Table S28:** Competing Risk Analysis for CV-related mortality at 5-years post AMI

| 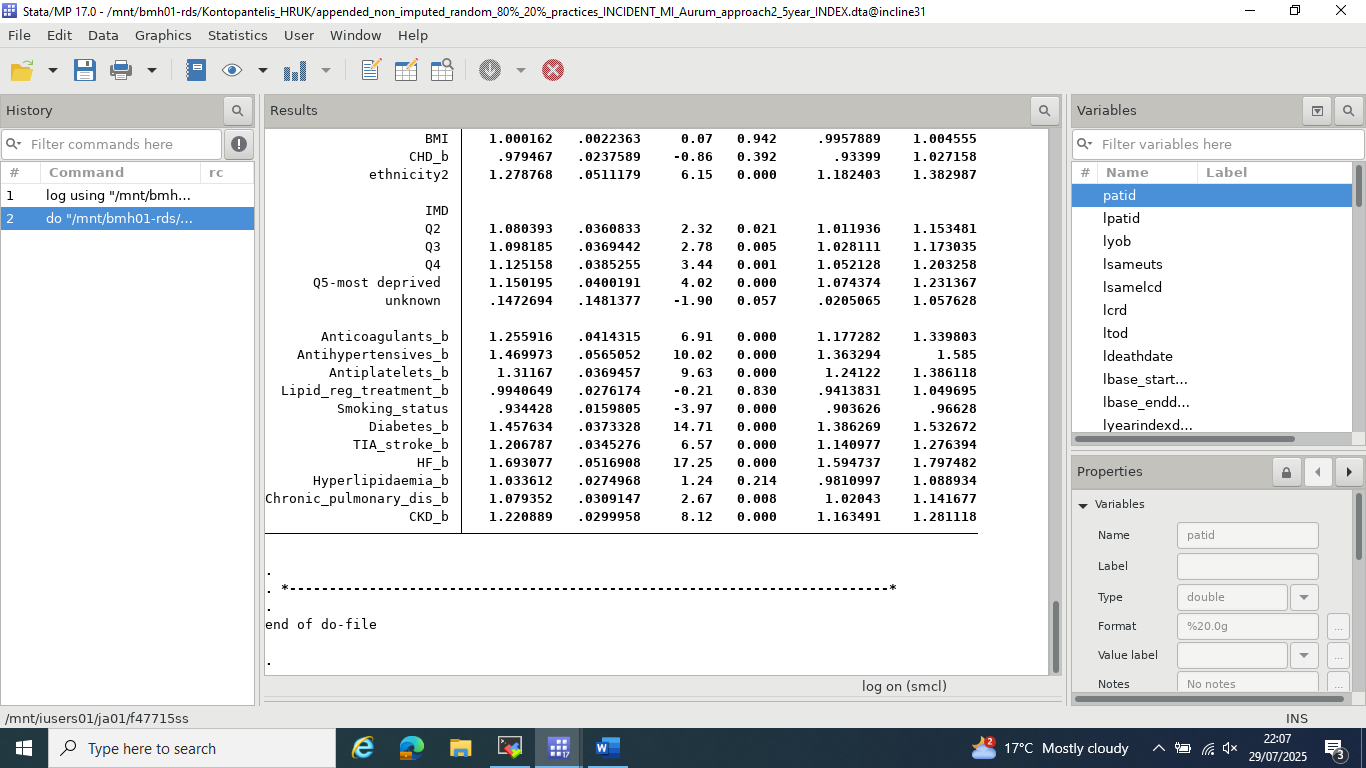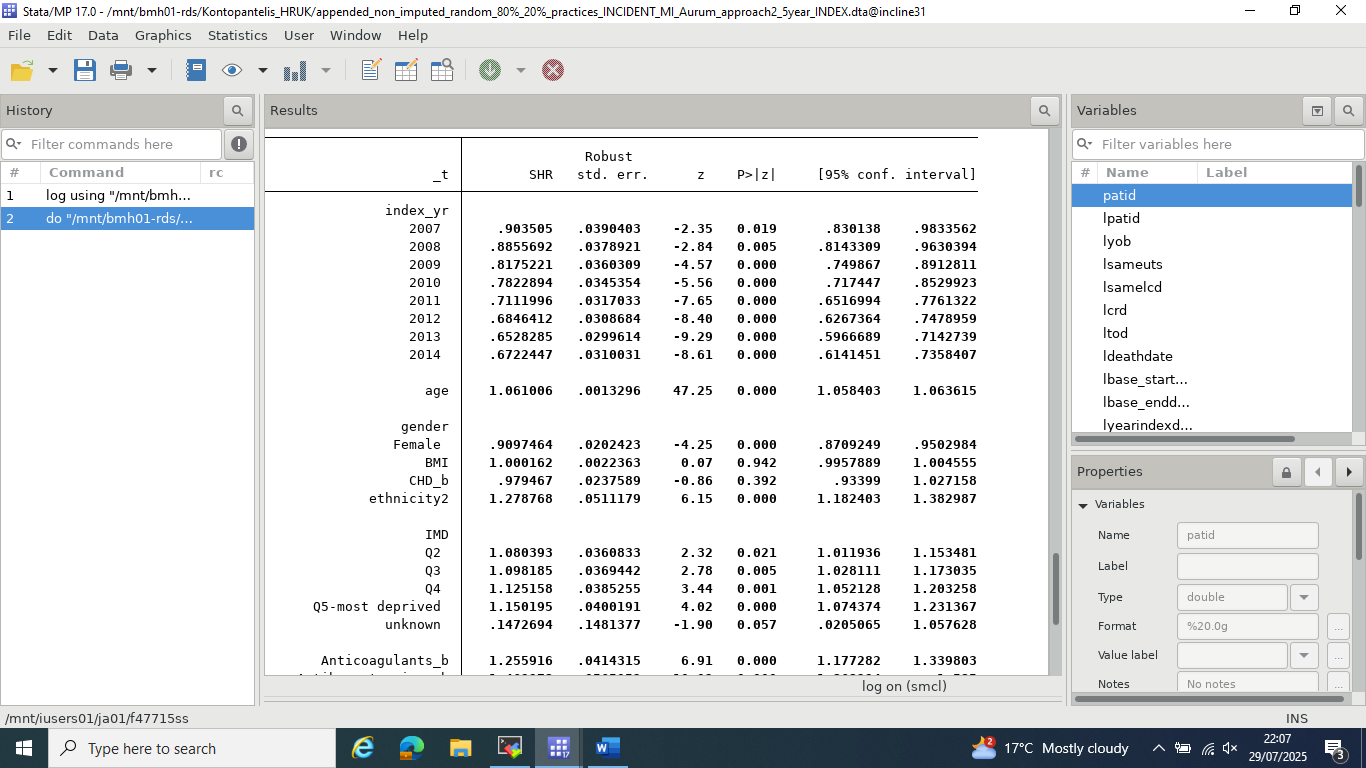 |
| --- |
| Competing Risks Analysis for CV-related mortality at 5-years with the influence of each variable shown. The competing risk was all-cause mortality. For sex, ethnicity, and IMD status, the references were male, White, and IMD Q1 respectively. CI: confidence interval. IMD: Index of Multiple Deprivation. SHR: Sub-distribution hazard ratio. Q: Quintile. Statistical significance at P<0.05. |

**Table S29:** Competing Risk Analysis for incident HF mortality at 5-years post AMI

| 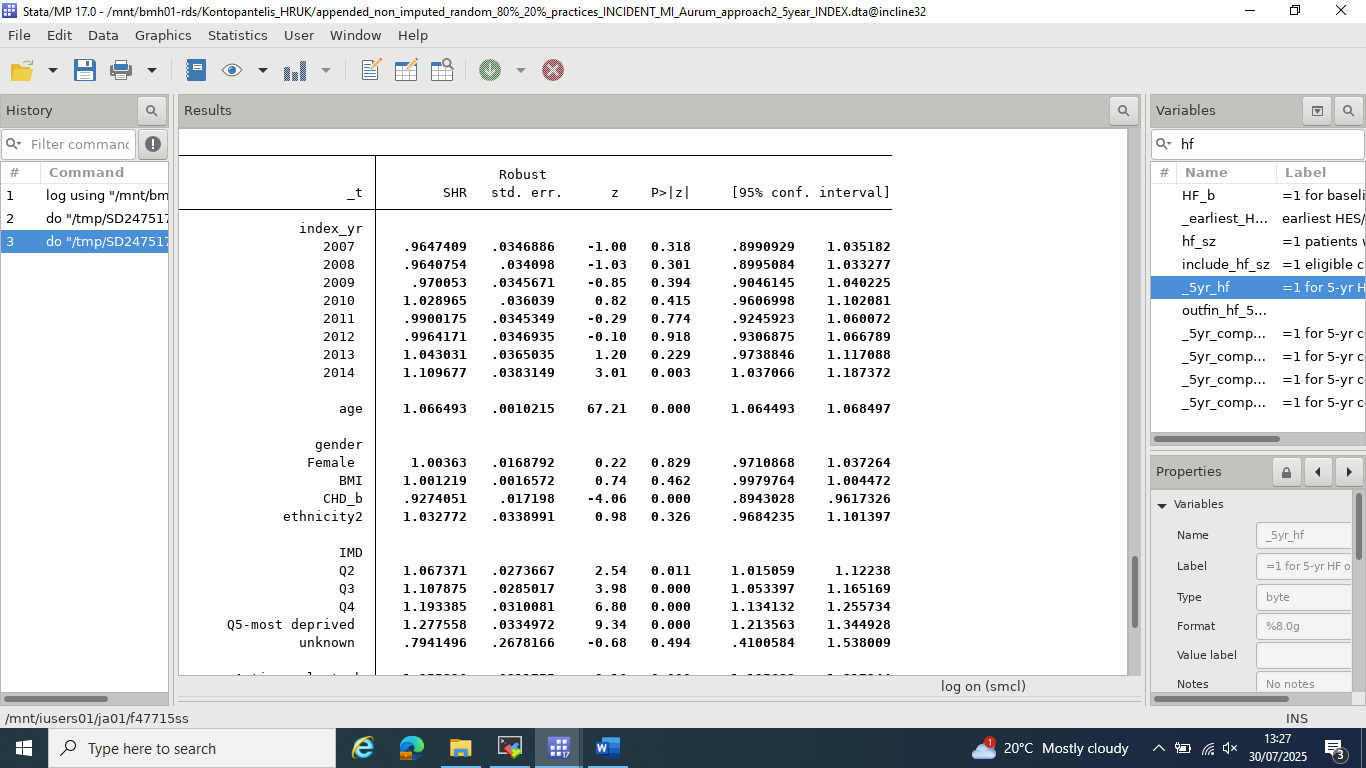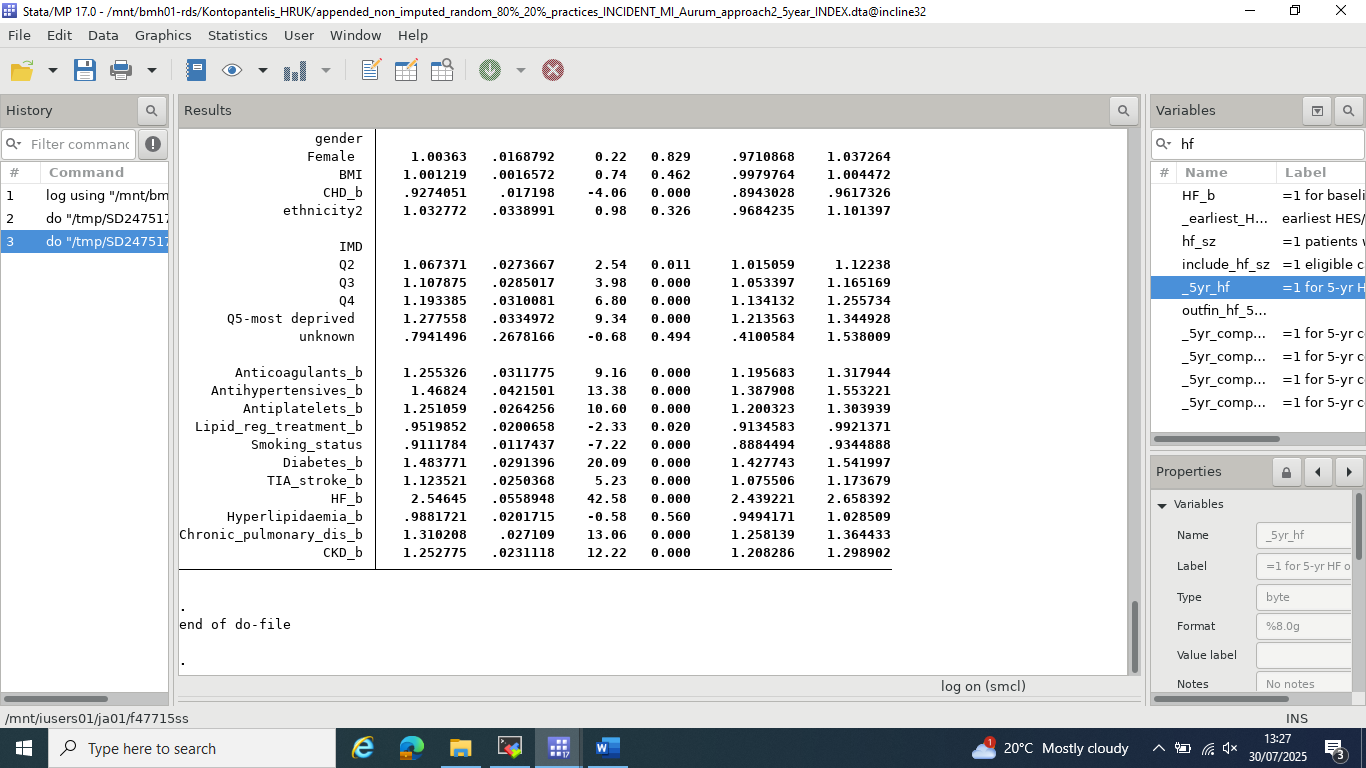 |
| --- |
| Competing Risks Analysis for incident HF at 5-years with the influence of each variable shown. The competing risk was all-cause mortality. For sex, ethnicity, and IMD status, the references were male, White, and IMD Q1 respectively. CI: confidence interval. IMD: Index of Multiple Deprivation. SHR: Sub-distribution hazard ratio. Q: Quintile. Statistical significance at P<0.05. |

**Table S30:** Competing Risk Analysis for incident CVA at 5-years post AMI

| 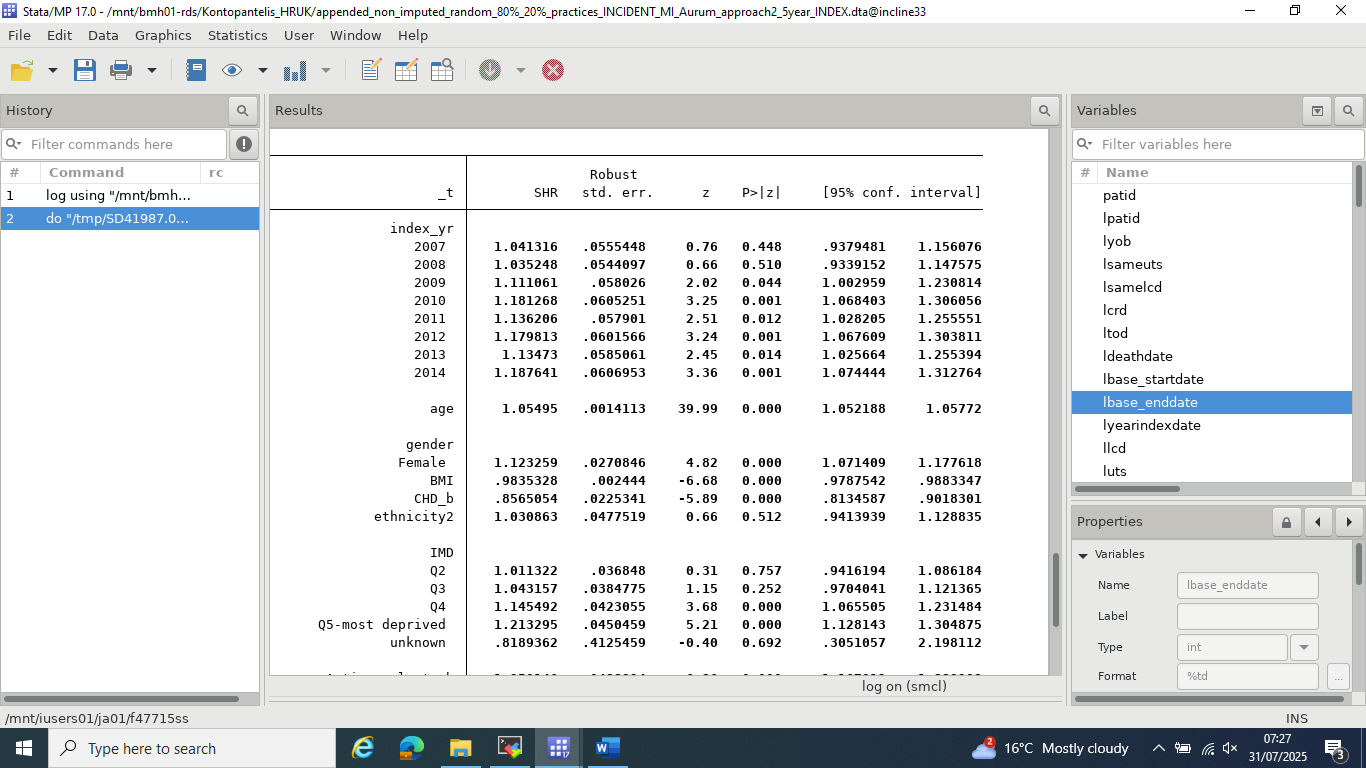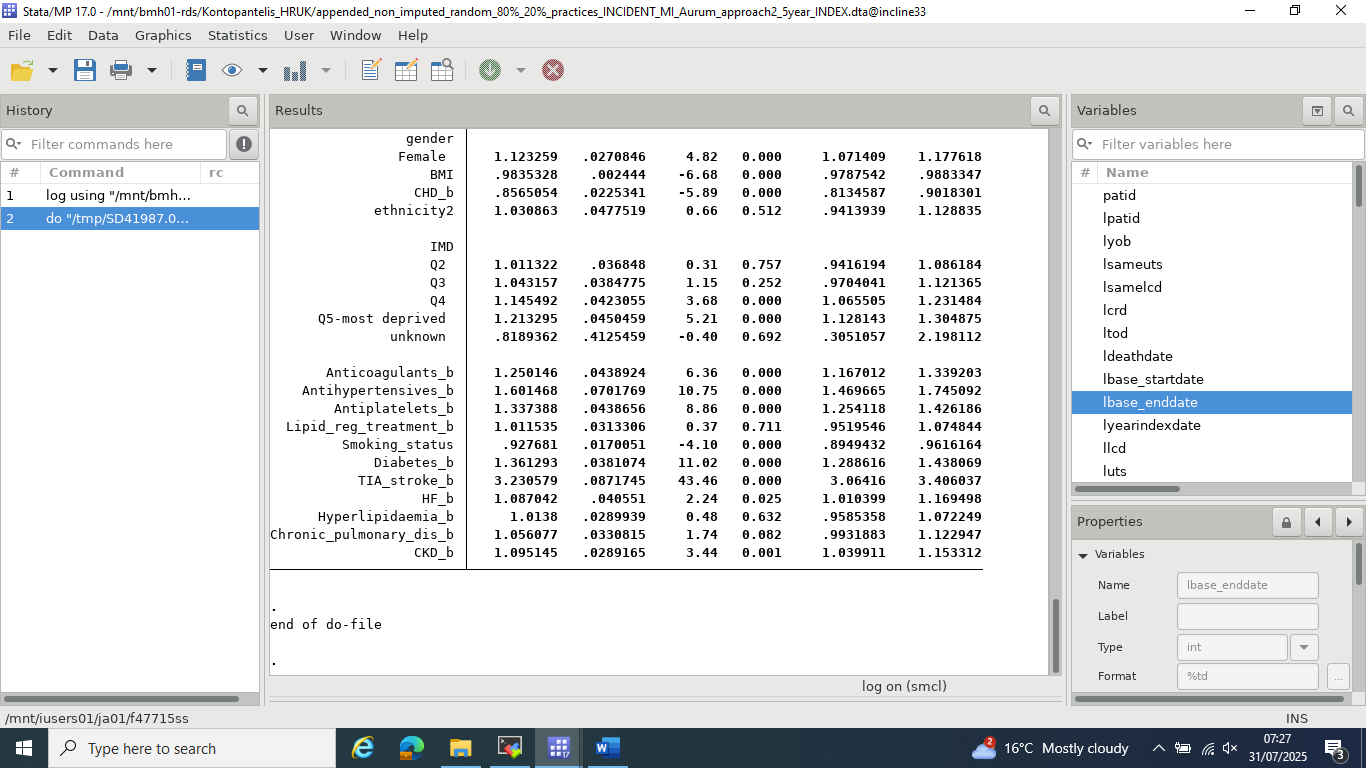 |
| --- |
| Competing Risks Analysis for CVA at 5-years with the influence of each variable shown. The competing risk was all-cause mortality. For sex, ethnicity, and IMD status, the references were male, White, and IMD Q1 respectively. CI: confidence interval. IMD: Index of Multiple Deprivation. SHR: Sub-distribution hazard ratio. Q: Quintile. Statistical significance at P<0.05. |

**Table S31:** Competing Risk Analysis for recurrent AMI at 5-years post AMI

| 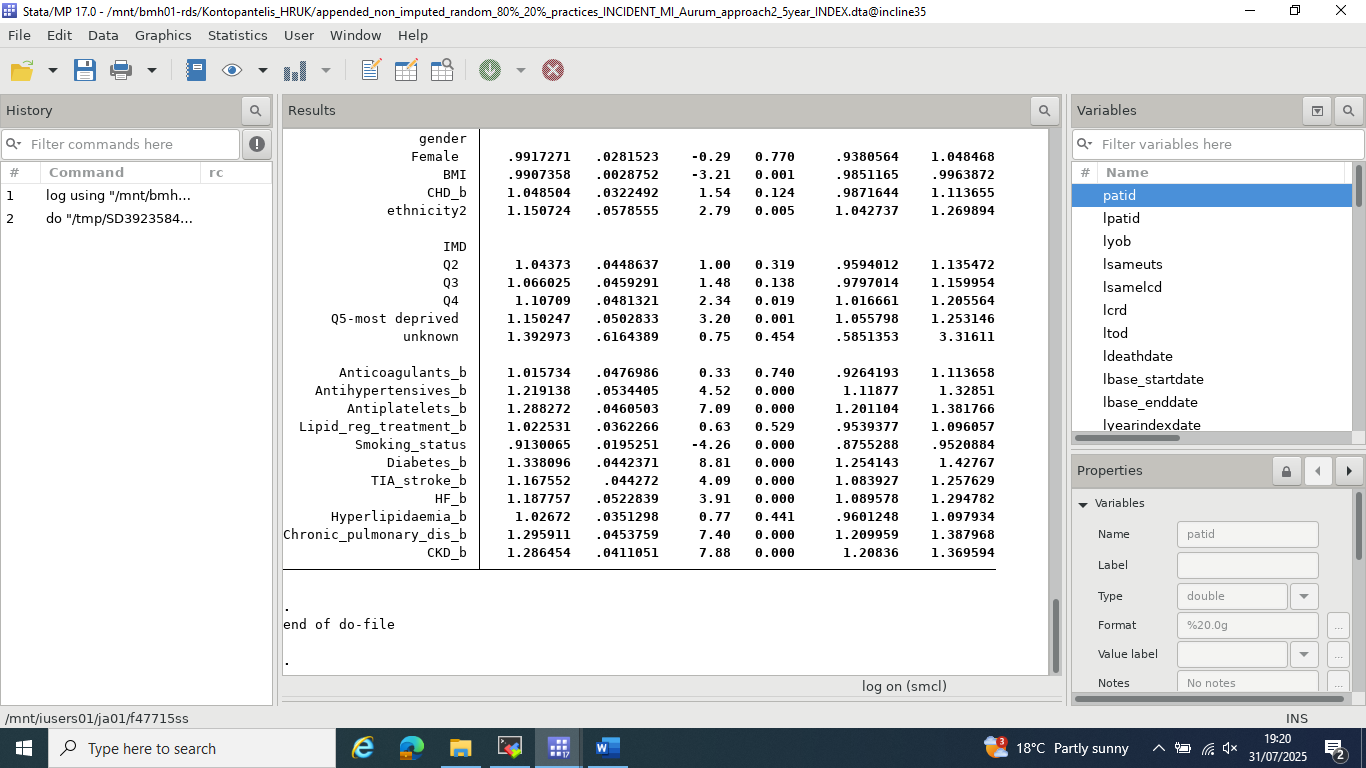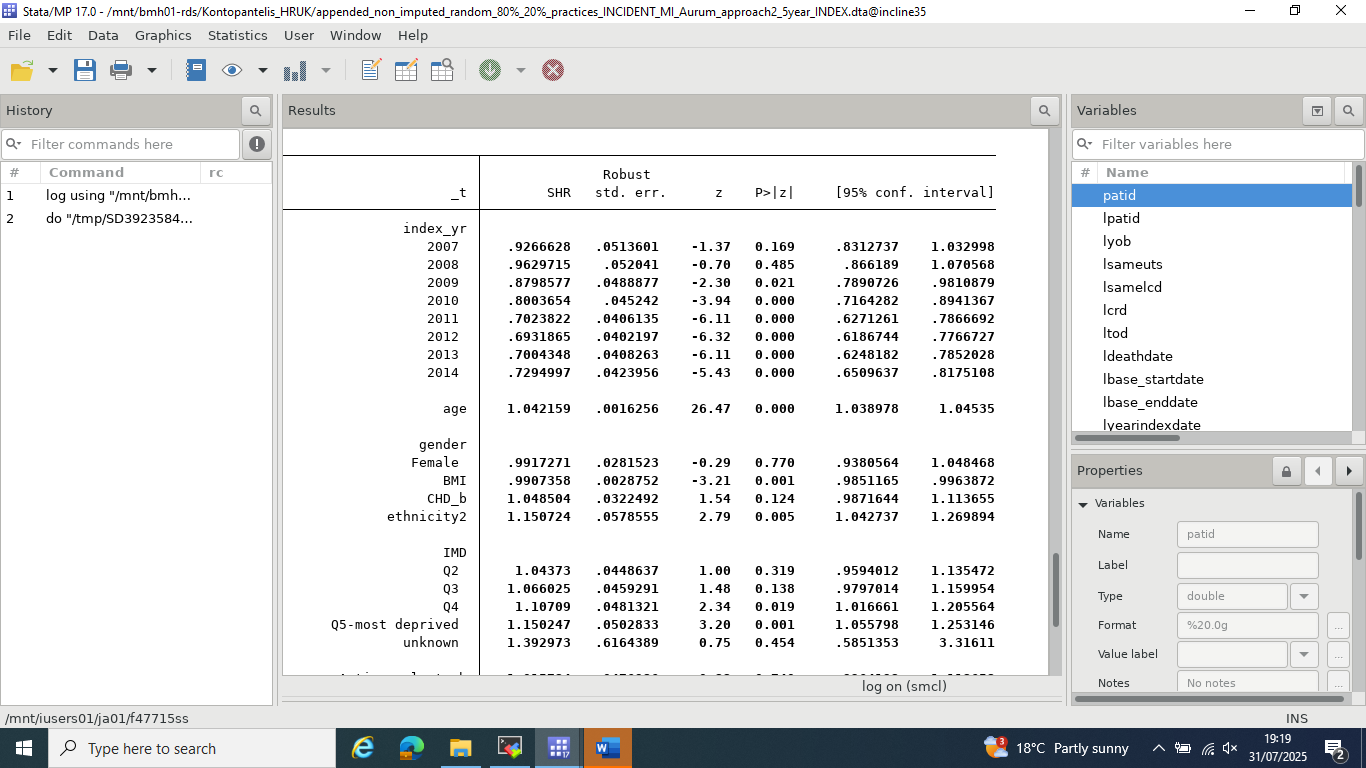 |
| --- |
| Competing Risks Analysis for recurrent AMI at 5-years with the influence of each variable shown. The competing risk was all-cause mortality. For sex, ethnicity, and IMD status, the references were male, White, and IMD Q1 respectively. CI: confidence interval. IMD: Index of Multiple Deprivation. SHR: Sub-distribution hazard ratio. Q: Quintile. Statistical significance at P<0.05. |
